# Myocardial pyruvate dehydrogenase kinase 4 drives sex-specific cardiac responses to endotoxemia

**DOI:** 10.1172/jci.insight.191649

**Published:** 2025-07-08

**Authors:** John Q. Yap, Azadeh Nikouee, Matthew Kim, Quan Cao, David J. Rademacher, Jessie E. Lau, Ananya Arora, Leila Y. Zou, Yuxiao Sun, Luke Szweda, Hesham Sadek, Sharon Elliot, Benjamin Roos, Marilyn K. Glassberg, Hong-Long Ji, Xiang Gao, Qunfeng Dong, Qun Sophia Zang

**Affiliations:** 1 Department of Surgery,; 2 Burn & Shock Trauma Research Institute,; 3 Department of Cell and Molecular Physiology,; 4 Cardiovascular Research Institute, and; 5 Department of Microbiology and Immunology, Loyola University Chicago, Stritch School of Medicine, Maywood, Illinois, USA.; 6 University of Missouri–Kansas City School of Medicine, Kansas City, Missouri, USA.; 7 Department of Surgery, University of Texas Southwestern Medical Center, Dallas, Texas, USA.; 8 Department of Medicine, Division of Cardiology, Sarver Heart Center, University of Arizona, Tucson, Arizona, USA.; 9 Department of Medicine and; 10 Center for Biomedical Informatics, Loyola University Chicago, Stritch School of Medicine, Maywood, Illinois, USA.

**Keywords:** Inflammation, Metabolism, Cardiovascular disease, Mitochondria, Molecular pathology

## Abstract

Males often experience worse cardiac outcomes than females in sepsis. This study identified pyruvate dehydrogenase kinase 4 (PDK4) as a key mediator of this disparity. PDK4 regulates glucose utilization by inhibiting pyruvate dehydrogenase (PDH) in mitochondria. In a mouse endotoxemia model, a sublethal dose of lipopolysaccharide (LPS, 5 mg/kg) significantly upregulated myocardial PDK4 and induced cardiac dysfunction in males but not females. Cardiac-specific PDK4 overexpression promoted this cardiac dysfunction in both sexes, whereas PDK4 knockout provided protection. In WT males, LPS reduced PDH activity and fatty acid oxidation (FAO) while increasing lactate levels, suggesting a shift toward glycolysis. These effects were exacerbated by PDK4 overexpression but attenuated by knockout. In females, metabolic changes were minimal, aside from reduced FAO in LPS-challenged females overexpressing PDK4. Additionally, a higher LPS dose (8 mg/kg) triggered cardiac dysfunction in females, accompanied by modest upregulation of PDK4, but without changes in PDH or lactate. Dichloroacetate (DCA), restraining PDK-mediated PDH inhibition, improved cardiac function in males but not females during endotoxemia. PDK4 overexpression also exacerbated cardiac mitochondrial damage, reduced mitophagy, and increased oxidative stress and inflammation during endotoxemia — effects that were prevented by PDK4 knockout. These findings suggest that PDK4 drives sex-specific cardiac responses in sepsis.

## Abstract 2

This study uncovers critical insights into mitochondrial signaling via PDK4 as a key contributor to sex-specific cardiac outcomes in response to sepsis or severe infections.

## Graphical abstract

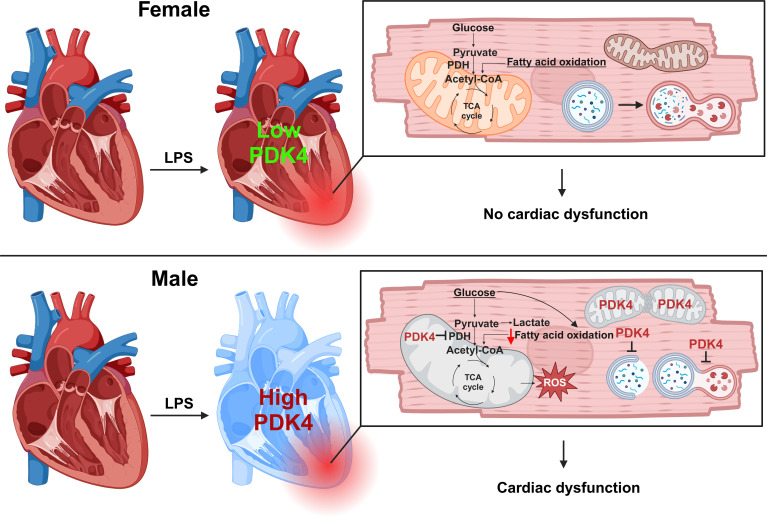

## Introduction

Sepsis is a life-threatening condition characterized by organ dysfunction due to a dysregulated host response to infection (1). This leads to systemic inflammation, metabolic dysfunction, and multiorgan failure (1–3). Among various complications of sepsis, sepsis-induced cardiomyopathy (SIC) is a major contributor to hemodynamic instability, impaired fluid imbalances, and inadequate oxygen delivery, which exacerbate systemic organ dysfunction and mortality (4). Despite advancements in antibiotic therapies and critical care techniques, the incidence of sepsis continues to rise (2, 5). Therefore, an in-depth understanding of the pathological mechanisms of sepsis and the development of new therapies are urgently needed. In this context, mechanisms of sex-specific outcomes during sepsis have been underexplored, and previous research has focused predominantly on male subjects. Recently, preclinical and clinical investigations revealed that sepsis outcomes differ between sexes, with females exhibiting less severe SIC compared with their male counterparts (4, 6, 7). These sex-specific differences are likely to involve both sex hormones (8, 9) and non-hormonal factors (10). A better understanding of the molecular mechanisms driving sex-specific outcomes during sepsis will pave the way for the future development of tailored, precise, and effective therapeutic strategies for sepsis patients.

Adverse outcomes in sepsis are frequently associated with disruptions in metabolic homeostasis and accumulation of metabolic intermediates from the breakdown of carbohydrates, lipids, and proteins (11–13). In the early phase of infection, the body’s physiological response demonstrates metabolic plasticity, which enables adaptation to short-term and mild metabolic disruptions. However, in the later stages of sepsis, the disruption of metabolic homeostasis becomes severe and irreversible, resulting in metabolic inflexibility (14, 15). At this stage, the body can no longer produce energy from commonly used metabolites and accumulates toxic substrates that eventually cause organ injury (11, 12, 16–18). Because mitochondria are primarily responsible for generating energy via glucose and fatty acid metabolism, there has been great interest in understanding how defects in mitochondria contribute to sepsis-induced metabolic inflexibility. Specifically, disruption of glucose metabolism due to decreased pyruvate dehydrogenase (PDH) activity has been observed in both clinical samples from sepsis patients (19) and animal models of sepsis (20, 21). PDH is the rate-limiting enzyme in glucose metabolism, converting pyruvate to acetyl coenzyme A (acetyl-CoA), and its activity is regulated by a family of pyruvate dehydrogenase kinases (PDK) within mitochondria (22). The expression of PDK isoforms appears tissue-specific, and PDK4 is one of the most prominent isoforms expressed in the heart (23). PDK4 is upregulated in cardiac tissue from male mice after cecal ligation and puncture–induced (CLP-induced) sepsis (21). Additionally, increased PDK4 was shown in serum samples from pediatric patients with SIC (24). These observations suggest that PDK4 may play a critical role in inducing metabolic inflexibility during SIC. However, whether PDK4 is involved in sex-specific outcomes during SIC has not been studied, and the mechanisms by which PDK4 disrupts mitochondrial properties remain unclear.

Research in our laboratory focuses on unraveling the molecular mechanisms of SIC, with a particular emphasis on the role of cardiac mitochondrial signaling. Our prior investigations showed that sepsis causes deficiencies in mitochondria, leading to the release of harmful danger-associated molecular patterns (DAMPs) that worsen inflammation within the heart (12, 25–27). We further unveiled a causative mechanism behind mitochondrial damage in septic hearts, which involves insufficient and maladaptive autophagy (28–31). Autophagy plays a critical role in maintaining cellular homeostasis under nutrient deprivation or energy stress. Here, we investigated how PDK4 modulates changes in cardiac metabolism and the sequential effects on mitochondrial properties and inflammation during SIC. To do so, we utilized mice with cardiac tissue–specific overexpression and knockout of PDK4. Bacterial endotoxin lipopolysaccharide (LPS) was injected intraperitoneally to simulate the acute inflammatory phase of sepsis. Our findings reveal that PDK4 drives sex-based differences in cardiac outcomes during acute inflammation by altering cardiac metabolism, mitochondrial structure and function, mitochondrial dynamics, mitophagy, oxidative stress, and inflammation. These new results improve our understanding of the molecular basis of metabolic dysfunction in SIC and its sex-specific mechanisms.

## Results

### Sex-specific cardiac performance and its association with myocardial PDK4 expression in response to endotoxemia.

**Figure 1:**
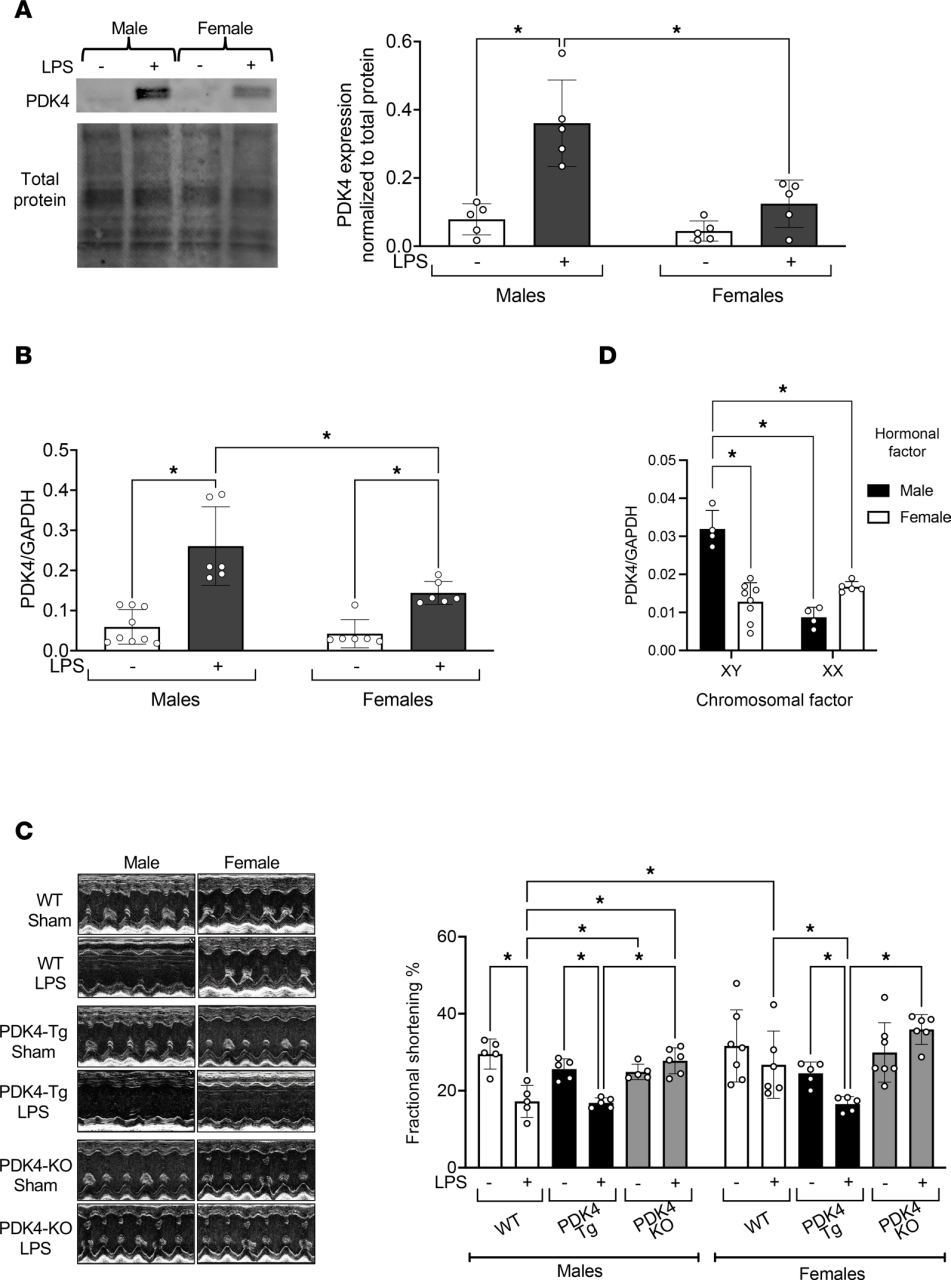
Association between PDK4 expression and cardiac performance in endotoxemia. In **A**–**C**, mice were given LPS challenge (5 mg/kg) or sham treatment and experiments were conducted 18 hours after treatment. (**A**) PDK4 levels in cardiac mitochondria of WT mice were examined by Western blot. Signals were quantified by densitometry, and results were normalized to total protein (*n* = 5). (**B**) PDK4 mRNA expression in cardiac tissue of WT mice was examined by qPCR. Results were normalized to GAPDH (*n* = 6–9). (**C**) The cardiac function of WT, PDK4-Tg, and PDK4-KO mice was evaluated by echocardiography. Values of fractional shortening were compared (*n* = 5–7). (**D**) PDK4 mRNA expression in cardiac tissue of FCG mice was examined by qPCR. Results were normalized to GAPDH (*n* = 4–8). The FCG mice have 4 genotypes, including genetic males (XY) with male gonads, genetic males (XY) with female gonads, genetic females (XX) with male gonads, and genetic females (XX) with female gonads. Data are presented as mean ± SEM and were analyzed by 2-way ANOVA. *P < 0.05.

We have previously established a mouse model of endotoxemia, in which we injected LPS intraperitoneally to trigger a severe acute inflammatory response, simulating the initial phase of sepsis (28). Our earlier studies showed that a sublethal dose of LPS challenge, 5 mg/kg, induces cardiac dysfunction in male mice, along with mitochondrial structural damage and functional impairments in the heart (28, 32, 33). The present study was designed to investigate the role of PDK4 in cardiac outcomes following endotoxemia. WT male and female mice were subjected to a 5 mg/kg intraperitoneal LPS challenge, while the sham groups received PBS. Heart tissue was harvested 18 hours after challenge, and PDK4 levels were analyzed in mitochondrial fractions using Western blotting (Figure 1A). Isolation of mitochondria was verified by assessing the mitochondrial marker protein voltage-dependent anion channel (VDAC) in cytosolic and mitochondrial fractions (Supplemental Figure 1A; supplemental material available online with this article; https://doi.org/10.1172/jci.insight.191649DS1). This LPS challenge increased PDK4 protein levels in cardiac mitochondria by approximately 4-fold and 2-fold in male and female mice, respectively (Figure 1A and Supplemental Figure 1B). Using real-time PCR quantification, we observed a similar sex-based difference in PDK4 gene expression in response to LPS; LPS-challenged males exhibited 2-fold higher PDK4 RNA levels compared to LPS-challenged females (Figure 1B).

In the same experimental setting, we assessed cardiac performance using echocardiography in mice 18 hours after challenge. Consistent with our previous findings (28, 32, 33), WT male mice exhibited compromised cardiac performance in response to the LPS challenge, as indicated by a significant decrease in fractional shortening (Figure 1C). However, female mice did not show any marked decline in cardiac function in response to the same LPS challenge, suggesting a relationship between PDK4 expression and cardiac function in response to acute inflammatory challenges.

To further determine whether PDK4 drives the sex-dependent cardiac outcomes during endotoxemia, we utilized 2 genetic mouse models: one with cardiac-specific PDK4 overexpression (PDK4-Tg) (34) and another with cardiac-specific PDK4 knockout (PDK4-KO) (35). Echocardiography assessment revealed that the LPS challenge impaired cardiac performance in both male and female PDK4-Tg mice, whereas PDK4 knockout protected cardiac function against the LPS challenge (Figure 1C). PDK4 overexpression and knockout were confirmed via Western blot analysis (Supplemental Figure 1C). Statistical analysis also revealed a marginal interaction (*P* = 0.053) between sex and PDK4 genotype under the LPS challenge, suggesting that the effects of PDK4 on cardiac function in response to LPS vary by sex. Taken together, these findings suggest that PDK4 may play a previously unrecognized role in mediating sex-specific cardiac performance in response to the acute inflammatory challenge induced by endotoxemia.

Lastly, we explored the mechanisms underlying the sex specificity of cardiac PDK4 expression using the Four Core Genotype (FCG) mouse model (36). This model uniquely differentiates the effects of sex chromosomes (XX versus XY) from those of gonadal sex (testes versus ovaries). In these mice, the Sry gene, which encodes the testis-determining factor, is translocated from the Y chromosome to an autosome. As a result, gonadal sex is no longer dictated by the presence of the Y chromosome. Thus, these FCG mice present four genotypes, including XX and XY gonadal males (XXM, XYM, respectively) and XX and XY gonadal females (XXF, XYF, respectively). We quantified PDK4 mRNA levels in the heart tissue of 17-month-old FCG mice. All mice underwent gonadectomy to eliminate any acute hormonal effects (Figure 1D). We found that XYM mice exhibited the highest PDK4 expression, suggesting that the Y chromosome and long-term exposure to gonadal hormones before gonadectomy may exert lasting organizational effects on gene expression and intrinsically regulate PDK4 expression in male hearts.

### PDK4 drives sex-based differences in cardiac metabolism during endotoxemia.

**Figure 2:**
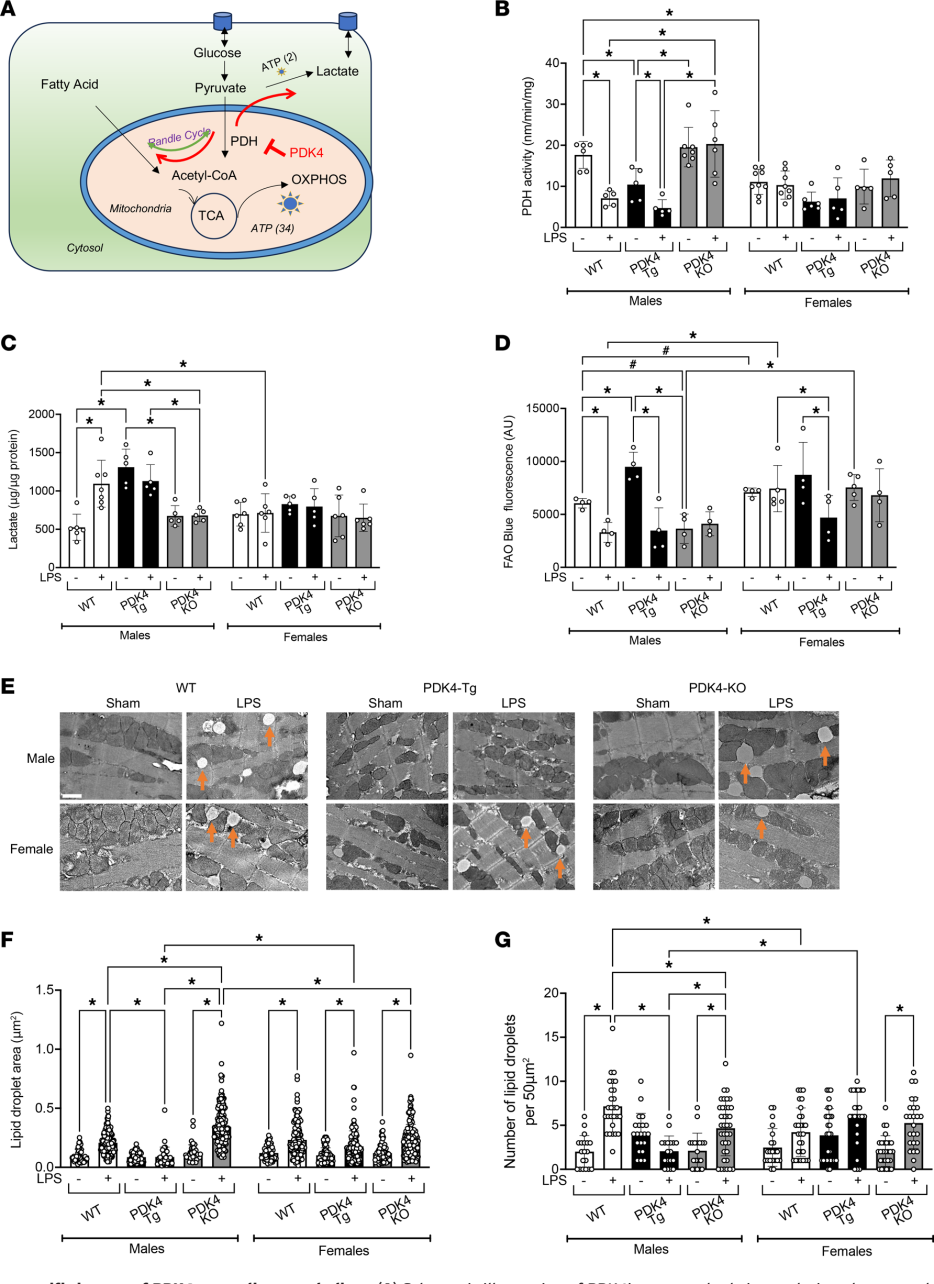
Sex-specific impact of PDK4 on cardiac metabolism. (**A**) Schematic illustration of PDK4’s proposed role in regulating glucose and fatty acid metabolism. The red arrows indicate metabolic shifts towards FAO in the mitochondria and overproduction of lactate in the cytoplasm due to PDH inhibition by PDK4. In **B**–**G**, WT, PDK4-Tg, and PDK4-KO mice were given LPS challenge (5 mg/kg) or sham treatment, and experiments were conducted 18 hours after treatment. (**B**) PDH activity and (**C**) lactate levels were measured in heart tissue lysates (*n* = 5–9 and *n* = 5–7, respectively). (**D**) FAO was assessed using FAOblue fluorescence in primary cardiomyocytes isolated from mice (*n* = 4–5 independent isolations per group). (**E**) Representative TEM images of cardiomyocytes with lipid droplets indicated by orange arrows. Images are representative of 3 independent isolations per group. Scale bar: 1 μm. (**F**) Lipid droplet area (*n* = 48–215 lipid droplets per group) and (**G**) number (*n* = 19–37 images per group) were quantified from TEM images using ImageJ software. Data in **F** and **G** are from 3 independent isolations per group. Data are presented as mean ± SEM and were analyzed using 2-way ANOVA (*P < 0.05) or Student’s *t* test ^(#^*P* < 0.05).

PDK4 plays a pivotal role in glucose and fatty acid metabolism by inhibiting the PDH complex in mitochondria (37, 38). As illustrated in Figure 2A, inhibition of PDH by PDK4 would decrease the conversion of pyruvate to acetyl-CoA, leading to an accumulation of pyruvate. Theoretically, this change in metabolism may cause a metabolic shift toward cytosolic glycolysis while increasing reliance on alternative fuel sources, such as fatty acids.

In WT male mice, the LPS challenge significantly decreased PDH activity by 61%, and increased lactate production by 114% (Figure 2, B and C). These results indicate a direct relationship between reduced PDH activity, increased lactate levels, and PDK4 upregulation in male hearts induced by acute inflammation. PDK4 overexpression in male mice significantly reduced PDH activity by 41% and increased lactate levels by 160% compared with WT male mice in the sham condition (Figure 2, B and C). The LPS challenge in PDK4-Tg males further decreased PDH activity. Conversely, PDK4 knockout prevented both the LPS-induced reduction in PDH activity and the LPS-induced increase in lactate production in male mice (Figure 2, B and C). These findings suggest that endotoxemia impairs the heart’s ability to utilize glucose for ATP production through upregulating PDK4 expression in males. This decrease in mitochondrial glucose oxidation also causes a metabolic shift toward cytosolic glycolysis.

Interestingly, we observed different results in female mice under the same experimental conditions. Sham-treated WT female mice exhibited a 29% lower PDH activity compared with their male counterparts (Figure 2B), suggesting sex-specific differences in cardiac glucose utilization between healthy males and females. Following the LPS challenge, no substantial changes in PDH activity or lactate levels were observed in WT female hearts. Furthermore, neither PDK4 overexpression nor PDK4 knockout significantly affected PDH activity or lactate levels (Figure 2, B and C). Statistical analysis further revealed a significant interaction between sex and PDK4 genotype for both PDH activity and lactate levels (*P* < 0.0047 and *P* < 0.012, respectively), indicating that the effects of PDK4 on these metabolic parameters differ between sexes. This analysis supports that PDK4-mediated effects on cardiac glucose metabolism are sex specific. Therefore, although cardiac-specific PDK4 overexpression in females decreased cardiac function (Figure 1C), this effect is likely mediated by mechanisms involving PDK4 other than altering glucose metabolism.

To examine the role of PDK4 in cardiac fatty acid metabolism, we measured fatty acid oxidation (FAO) levels using an FAO-specific fluorescent indicator in primary adult cardiomyocytes. Validation of the experimental protocol is shown in Supplemental Figure 2. We compared FAO levels in cardiomyocytes isolated from WT, PDK4-Tg, and PDK4-KO mice subjected to either the LPS challenge or sham treatment. In WT males, the LPS challenge caused a significant 42% reduction in FAO (Figure 2D). This result is consistent with previous findings demonstrating that sepsis compromises FAO due to disrupted mitochondrial health and biogenesis (39–41). Compared with sham-treated WT males, sham-treated PDK4-Tg males exhibited a 58% increase in FAO (Figure 2D), supporting the notion that PDK4 promotes a shift in fuel preference from glucose metabolism to fatty acid utilization under normal physiological conditions. This result is consistent with the concept of the Randle cycle, in which a reciprocal relationship between glucose and fatty acid metabolism maintains metabolic flexibility (42). However, cardiomyocytes isolated from male PDK4-Tg mice challenged with LPS exhibited a more significant reduction in FAO levels, with a decrease of approximately 63% (Figure 2D), suggesting that PDK4 overexpression might render cardiomyocytes more vulnerable to impairments in mitochondrial metabolism under stress conditions. In contrast, FAO levels in cardiomyocytes from sham-treated PDK4-KO male mice were decreased compared with those in sham-treated WT males. This result is consistent with our PDH activity and lactate level findings (Figure 2, B and C) and suggests that blunting PDK4 signaling increases glucose metabolism and reduces the reliance on fatty acid metabolism. Furthermore, FAO in PDK4-KO males was unchanged in response to LPS challenge (Figure 2D), suggesting that PDK4 knockout confers resistance to the detrimental effects of LPS.

For the female phenotypes, cardiomyocytes from sham-treated WT female mice exhibited 17% higher FAO levels compared with cardiomyocytes isolated from sham-treated WT males (Figure 2D), showing sex-specific differences in fatty acid utilization under physiological conditions. Additionally, the LPS challenge in WT females had little impact on FAO levels, suggesting that mitochondrial function was largely unaffected in these cells. In contrast with the result observed in male mice, there was no difference in FAO levels between cardiomyocytes from sham-treated PDK4-Tg female mice and cardiomyocytes from sham-treated WT female mice. However, the LPS challenge in PDK4-Tg female mice caused a significant decrease in FAO levels (Figure 2D), similar to the reduction observed in LPS-treated PDK4-Tg males. These results suggest that PDK4 overexpression induces impairments in mitochondrial function, rendering them unable to sustain FAO in both male and female hearts under acute inflammatory conditions. Lastly, no LPS-associated changes in FAO were detected in cardiomyocytes from PDK4-KO female mice (Figure 2D). Notably, cardiomyocytes from female PDK4-KO hearts exhibited approximately double the FAO levels of those from male PDK4-KO hearts, further highlighting the sex-specific differences in cardiac metabolism between males and females. This sex-specific variation of FAO level across PDK4 genotypes was further supported by a significant interaction between sex and PDK4 (*P* = 0.045).

To further assess fatty acid metabolism, we performed quantitative analyses of lipid droplets in the myocardium. Using transmission electron microscopy (TEM), we visualized lipid droplets in primary cardiomyocytes isolated from male and female WT, PDK4-Tg, and PDK4-KO mice. Lipid droplet properties were then characterized by quantifying their population (number) and individual size (area). Previous studies have shown that systemic inflammation increases circulating free fatty acid levels, leading to a potential increase in fatty acid uptake by cardiomyocytes (43, 44). These fatty acids can subsequently be utilized by mitochondria for energy production, and, if not, stored as lipid droplets (45–47). Consistent with these findings, we observed that changes in lipid droplet properties depend on systemic influences. Notably, in vitro LPS treatment of isolated cardiomyocytes did not alter lipid droplet formation (Supplemental Figure 3). This initial analysis underscores the importance of the in vivo LPS challenge–associated systemic effects on lipid droplet dynamics and their physiological relevance in fatty acid uptake.

Therefore, the analysis of lipid droplets was performed in cardiomyocytes isolated from LPS-challenged or sham-treated mice; these data are given in Figure 2, E–G and Supplemental Figure 4. Cardiomyocytes from LPS-challenged WT male mice showed significant increases in both the size and number of lipid droplets compared with their sham-treated counterparts, consistent with our recent report (48). Interestingly, PDK4 overexpression abolished these LPS-induced changes in lipid droplet properties. In contrast, cardiomyocytes from LPS-treated PDK4-KO male mice showed a significant increase in lipid droplet size but a slight decrease in lipid droplet number. In cardiomyocytes from WT females, LPS challenge significantly increased lipid droplet size, with only a marginal effect on their number. Cardiomyocytes from LPS-challenged PDK4-Tg and PDK4-KO females showed similar increases in lipid droplet area and number compared to LPS-treated WT females. Collectively, these data suggest that lipid droplet formation may serve as an adaptive mechanism to support cardiomyocyte survival during the metabolic stress induced by acute inflammation, as accumulating free fatty acids in the cytoplasm causes lipotoxicity (44, 49). In males, particularly, this protective response appears to be suppressed by PDK4 overexpression but promoted by PDK4 knockout, highlighting PDK4 as a key regulator of fatty acid homeostasis and lipid droplet dynamics in cardiomyocytes during inflammatory challenges.

### PDK4 upregulation disrupts mitochondrial structure during endotoxemia.

**Figure 3:**
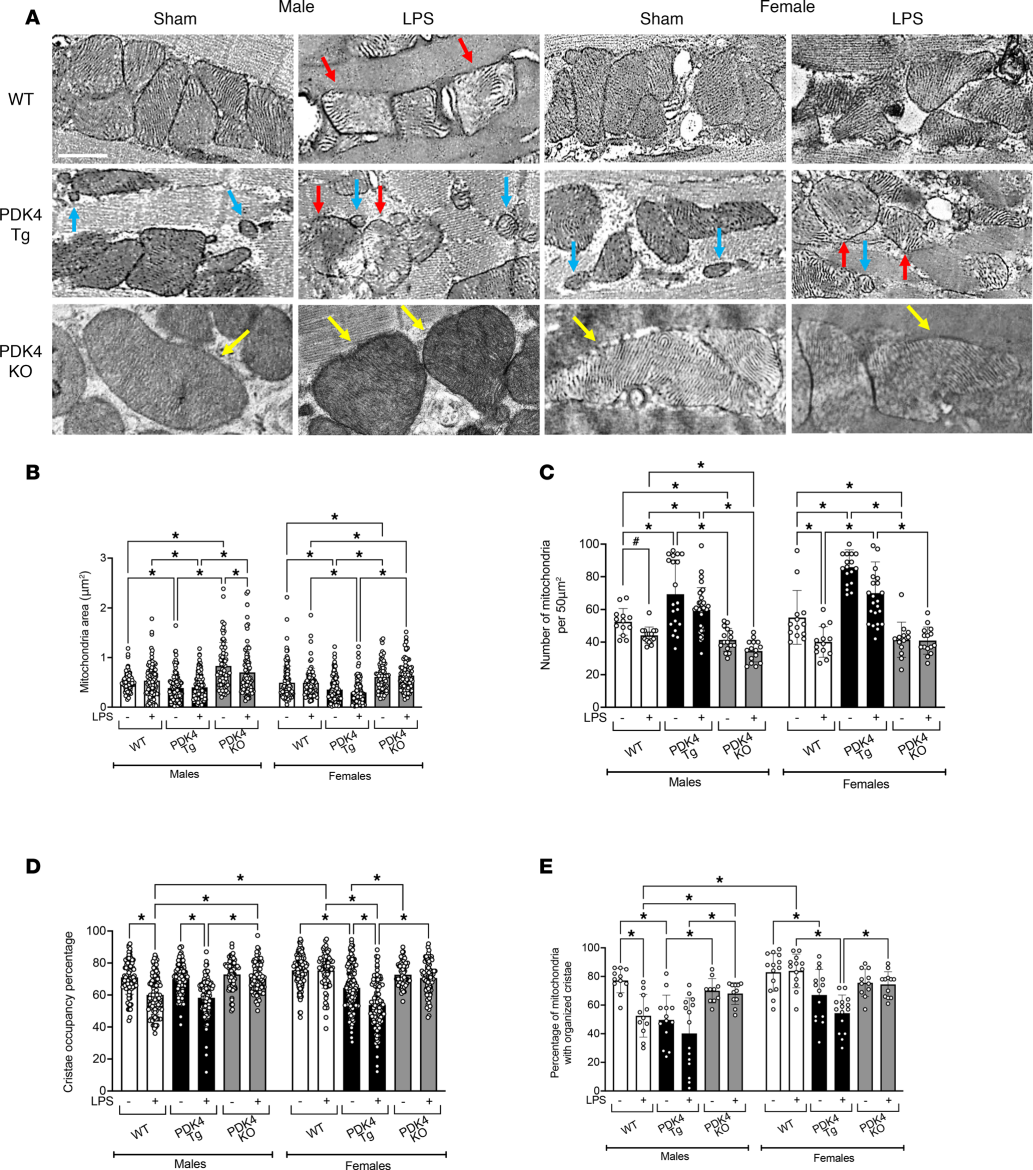
PDK4 impact on mitochondrial structure and morphology in cardiomyocytes. WT, PDK4-Tg, and PDK4-KO mice were given LPS challenge (5 mg/kg) or sham treatment, and cardiomyocytes were isolated 18 hours after treatment. (**A**) Representative TEM images of cardiomyocytes. Red arrows depict damaged mitochondria. Blue arrows depict mitochondrial fragmentation. Yellow arrows depict large mitochondria. Images are representative of 3 independent isolations per group. Scale bar: 1 μm. (**B**) Individual mitochondrial area in mm^2^ (*n* = 67–130 mitochondria per group), (**C**) number of mitochondria per 50 μm^2^ (*n* = 13–34 images per group), (**D**) percentage of cristae occupancy relative to total mitochondria area (*n* = 67–130 mitochondria per group), and (**E**) percentage of mitochondria with disorganized cristae relative to the number of mitochondria per 50 mm^2^ (*n* = 11–15 images per group) were quantified using Image J software based on TEM images. Data in **B**–**E** are from 3 independent isolations per group. Data are presented as mean ± SEM and were analyzed using 2-way ANOVA (*P < 0.05) or Student’s *t* test (^#^*P* < 0.05).

The mitochondrial structure serves as a key indicator of both mitochondrial function and overall mitochondrial health. Using TEM, we analyzed parameters of mitochondrial structure in isolated primary cardiomyocytes from male and female WT, PDK4-Tg, and PDK4-KO mice with LPS challenge or sham treatment. Representative TEM images of cardiomyocytes from each group are shown in Figure 3A. The parameters measured include mitochondria size (area), mitochondria population (number), inner cristae density (percentage of the mitochondria occupied by cristae), and mitochondria cristae integrity (percentage of mitochondria with organized cristae), which are shown in Figure 3, B–E.

In cardiomyocytes isolated from both male and female WT mice, LPS challenge decreased mitochondria number without affecting mitochondrial area (Figure 3, B and C). This is consistent with previous findings showing that LPS decreases mitochondria mass in the myocardium due to structural damage in mitochondria and increased mitophagy (28). Importantly, the LPS challenge in males significantly decreased cristae occupancy by 14%, as well as the percentage of mitochondria with organized cristae by 31%. However, the LPS challenge did not significantly impact either of these parameters in females, demonstrating sex-specific differences in maintaining mitochondria structural integrity in the heart during acute inflammation. Interestingly, PDK4 overexpression induced a significant increase in mitochondria number, but a decrease in mitochondria area in cardiomyocytes isolated from both males and females, indicative of mitochondria fission. In Figure 3A, the smaller, fragmented mitochondria are indicated by the blue arrows. The LPS challenge decreased cristae occupancy and disrupted cristae organization in cardiomyocytes from PDK4-Tg mice, indicating damaged mitochondrial structure. However, cardiomyocytes from PDK4-KO mice exhibited the opposite phenotype, in which a reduced number of mitochondria and an increase in mitochondrial area were observed, indicative of mitochondrial fusion (Figure 3, B and C). Our observation suggests that PDK4 may play a critical role in regulating mitochondria fission-fusion dynamics in the heart during acute inflammation. This result is consistent with a recently published study demonstrating the noncanonical role of PDK4 in promoting mitochondrial fission, independent of its well-established function in metabolic regulation (50). Importantly, PDK4 knockout in males significantly increased cristae occupancy and ameliorated cristae disorganization in response to the LPS challenge (Figure 3, D and E), protecting the myocardial mitochondria against acute inflammation-induced pathological damage. Together, these results show that PDK4 upregulation disrupts mitochondrial structure and has additional regulatory roles in mitochondrial dynamics in the heart during endotoxemia.

### PDK4 upregulation impairs mitochondria health and induces oxidative stress during endotoxemia.

**Figure 4:**
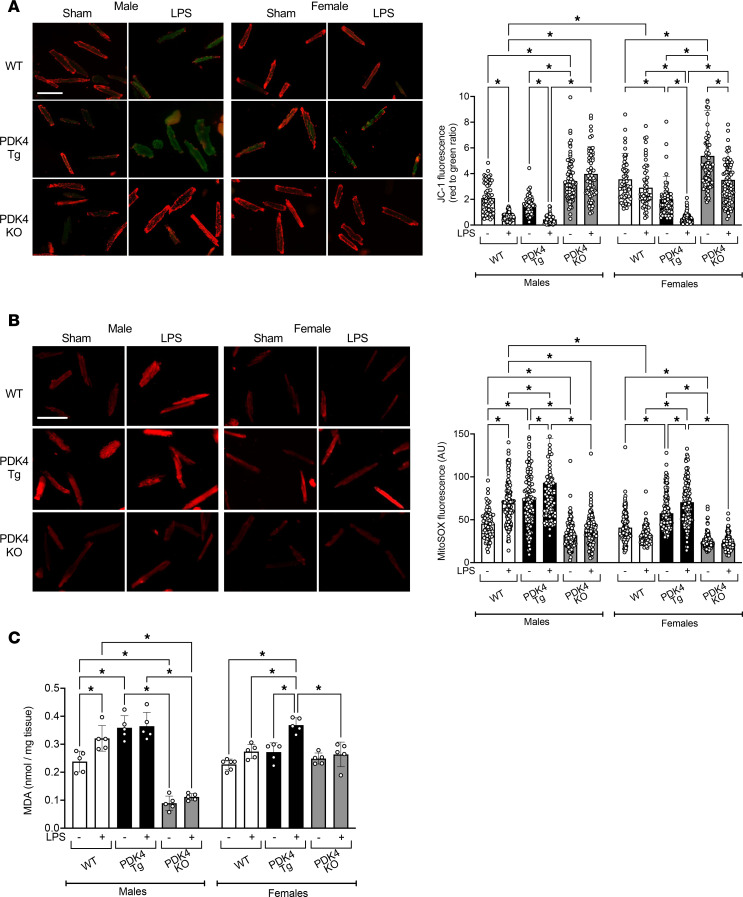
PDK4 impact on mitochondrial health and ROS production in the heart. WT, PDK4-Tg, and PDK4-KO mice were given LPS challenge (5 mg/kg) or sham treatment, and experiments were conducted 18 hours after treatment. (**A**) Mitochondrial membrane potential measured by of JC-1 fluorescence in isolated cardiomyocytes (*n* = 54–101 cardiomyocytes per group). (**B**) Mitochondrial superoxide production measured by MitoSOX Red fluorescence in isolated cardiomyocytes (*n* = 73–185 cardiomyocytes per group). Scale bar (**A** and **B**): 100 μm. Images and analyses are representative of 3 independent isolations per group. (**C**) MDA levels measured in heart tissue lysates (*n* = 5–6). Data are presented as mean ± SEM and were analyzed using 2-way ANOVA. *P < 0.05.

Since mitochondrial structure is closely linked to mitochondrial health, we examined how PDK4 influences mitochondrial membrane potential in primary cardiomyocytes isolated from male and female WT, PDK4-Tg, and PDK4-KO mice with the LPS challenge or sham treatment. Using JC-1, a fluorescent indicator of mitochondrial membrane potential, we observed that the LPS challenge in WT male mice resulted in a 71% reduction in mitochondrial membrane potential in cardiomyocytes. However, the LPS effect on the mitochondrial membrane potential from female cardiomyocytes was minimal (Figure 4A). Aligning with its pathological effects on mitochondrial structure (Figure 3), PDK4 overexpression exacerbated the LPS-induced decrease in mitochondrial membrane potential in both sexes. Conversely, PDK4 knockout significantly elevated mitochondrial membrane potential, providing resistance against the LPS challenge (Figure 4A). In the female PDK4-KO group, although an LPS-induced reduction in mitochondrial membrane potential was observed, the membrane potential remained comparable to that of WT females with sham treatment, suggesting that the mitochondria in this group could still maintain their functionality despite the LPS challenge.

Additionally, we evaluated mitochondrial damage by measuring the production of reactive oxygen species (ROS). ROS production in isolated cardiomyocytes was assessed using MitoSOX Red, a fluorescent dye that specifically detects mitochondrial superoxide production in live cells. We observed that the LPS challenge caused a significant increase in superoxide levels in cardiomyocytes from male mice by 56%, but its impact on female mice was minimal (Figure 4B). This result is consistent with the current literature, which suggests that females have increased resistance to ROS production compared with males (51). In PDK4-Tg mice under sham conditions, PDK4 overexpression increased ROS production by 67% in males and 40% in females, and LPS challenge further augmented ROS levels in both sexes (Figure 4B). In contrast, PDK4 knockout suppressed ROS production under all conditions (Figure 4B).

Furthermore, by measuring methylenedioxyamphetamine (MDA) in tissue lysates, we evaluated oxidative stress in heart tissue from WT, PDK4-Tg, and PDK4-KO mice subjected to either the LPS challenge or sham treatment (Figure 4C). MDA is a harmful byproduct of lipid peroxidation by ROS, and these lipid radicals can cause membrane damage, loss of membrane integrity, and impaired cellular function (52, 53). Consistent with results obtained in isolated cardiomyocytes, the LPS challenge significantly increased MDA levels by 33% in male hearts but had little effect on MDA levels in female hearts. Cardiac-specific PDK4 overexpression in males significantly elevated lipid peroxidation by 50% in both sham-treated and LPS-challenged mice. Although the LPS challenge did not affect MDA levels in WT female hearts, PDK4 overexpression in female hearts triggered an LPS-induced increase in MDA by 37%. PDK4-KO significantly limited lipid peroxidation in the heart, especially in males. In addition, statistical analysis revealed a significant 3-way interaction among sex, PDK4 genotype, and LPS challenge (*P* = 0.0099), indicating that LPS-induced oxidative stress was affected by both sex and PDK4 expression. Taken together, these results suggest that PDK4 upregulation promoted cardiac dysfunction by impairing mitochondrial health and increasing oxidative damage in the myocardium during endotoxemia.

### PDK4 upregulation disrupts mitophagy during endotoxemia.

**Figure 5:**
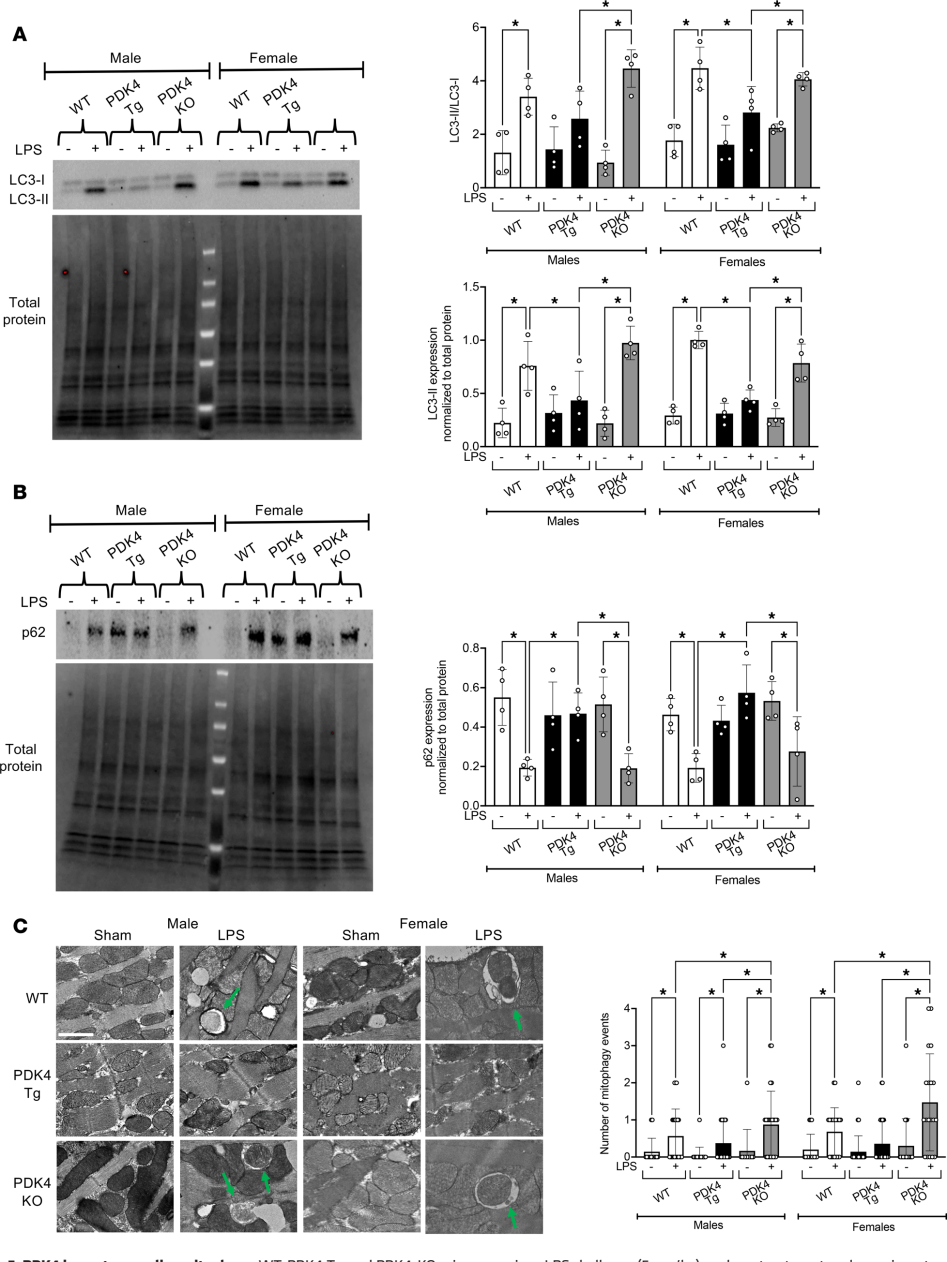
PDK4 impact on cardiac mitophagy. WT, PDK4-Tg, and PDK4-KO mice were given LPS challenge (5 mg/kg) or sham treatment,and experiments were performed 18 hours after treatment. (**A**) LC3II/LC3I and (**B**) p62 levels in crude mitochondrial fractions isolated from heart tissue were analyzed by Western blots. Densitometric quantification was performed with normalization to total protein (*n* = 4). (**C**) Representative TEM images of cardiomyocytes. Green arrows indicate mitophagy events. Scale bar: 1 μm. Images are representative of 3 independent isolations per group. The number of mitophagy events was quantified per 50 μm² (*n* = 12–35 images from 3 independent isolations per group). Data are presented as mean ± SEM and were analyzed using 2-way ANOVA. *P < 0.05.

Recent studies have demonstrated that PDK4 upregulation disrupts autophagy (54, 55). Given our findings that PDK4 alters mitochondrial structure and function, we investigated its role in mitophagy, a subset of autophagy crucial for cellular repair. The generation of LC3II and an increased LC3II/LC3I ratio indicate the formation of autophagosomes and mitophagosomes during the initial phase of autophagy, whereas a decrease in p62 serves as a marker for downstream degradation through lysosomes, showing the late stage of autophagic flux (56, 57). To assess mitophagy, we used Western blotting to measure the conversion of LC3I to LC3II and p62 levels in crude mitochondrial fractions, in which mitochondria and their associated proteins were kept intact (Figure 5 and Supplemental Figure 5). Our results showed that in both male and female WT mice, the LPS challenge increased LC3II levels, as well as the LC3II/LC3I ratio (Figure 5A), while reducing p62 levels (Figure 5B), indicating an enhanced mitophagy response. Compared with the LPS-challenged WT mice, LPS-challenged PDK4-Tg male and female mice displayed reduced LC3II levels, reduced LC3II/LC3I ratio (Figure 5A), and increased p62 levels (Figure 5B), indicating disrupted mitophagy. Conversely, PDK4-KO mice showed a robust LPS-induced mitophagy response, as evidenced by the increased LC3II levels, increased LC3II/LC3I ratio (Figure 5A), and decreased p62 levels compared with the LPS-induced effects on WT mice (Figure 5B).

To further evaluate mitophagy, we used TEM to quantify mitophagy events in cardiomyocytes isolated from WT, PDK4-Tg, and PDK4-KO mice with the sham treatment or the LPS challenge (Figure 5C). Consistent with our Western blotting analysis, we observed that the LPS challenge increased the number of mitophagy events in cells from both WT males and females. However, this response was attenuated in PDK4-Tg mice but significantly enhanced in PDK4-KO mice, regardless of sex (Figure 5C). Together, these results suggest that PDK4 upregulation contributes to cardiac dysfunction by disrupting mitophagy. Our observation is consistent with previous reports in the literature, which showed PDK4-mediated inhibition of autophagy in vascular smooth muscle cells (54) and breast cancer cells (55).

### PDK4 upregulation mediates cardiac inflammation during endotoxemia.

**Figure 6:**
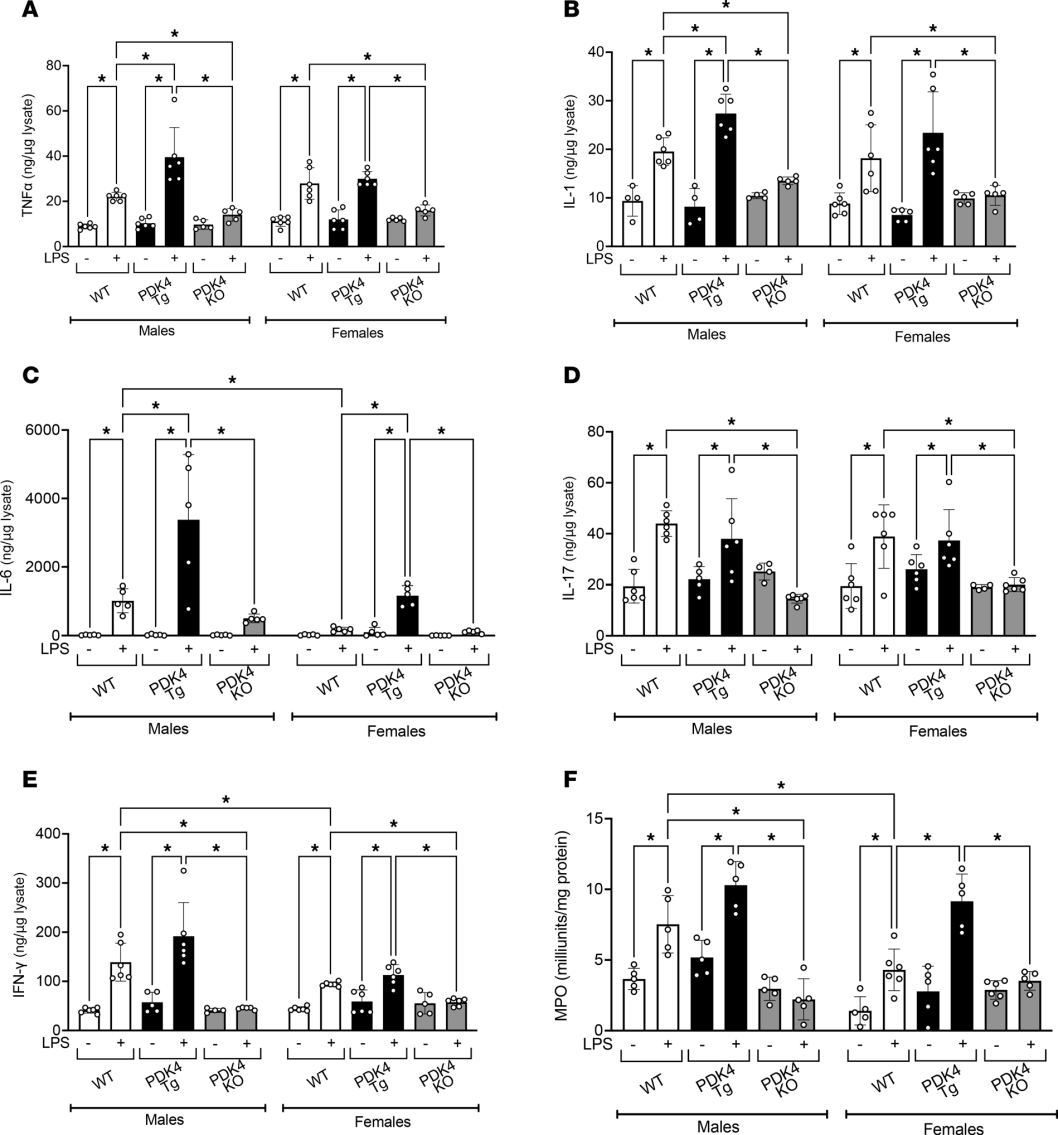
PDK4 impact on cardiac inflammation. WT, PDK4-Tg, and PDK4-KO mice were given LPS challenge (5 mg/kg) or sham treatment, and experiments were performed 18 hours after treatment. Levels of (**A**) TNF-α (*n* = 5–6), (**B**) IL-1 (*n* = 4-6), (**C**) IL-6 (*n* = 5), (**D**) IL-17 (*n* = 4-6), and (**E**) INF-γ (*n* = 4-6) were measured in cardiac tissue lysates by ELISA. Results were normalized by total protein. (**F**) MPO activity, a marker of neutrophil infiltration, was measured in cardiac tissue lysates (*n* = 5–6). Data are presented as mean ± SEM and were analyzed using 2-way ANOVA. *P < 0.05.

During sepsis, cardiac inflammation activates proinflammatory signaling pathways, resulting in immune cell recruitment to the site of inflammation and increased expression of proinflammatory cytokines, adhesion molecules, and chemokines (58). Therefore, we measured cytokine levels in heart tissue from WT, PDK4-Tg, and PDK4-KO mice with the LPS challenge or sham treatment (Figure 6, A–E). In WT males, the LPS challenge significantly increased levels of proinflammatory cytokines, including tumor necrosis factor-α (TNF-α), IL-1, IL-6, IL-17, and INF-γ. Notably, among these cytokines, IL-6 levels showed the most dramatic increase, with concentrations increasing significantly from 17 ng/mg lysate to 1,018 ng/mg lysate, an approximately 60-fold elevation. Overexpression of PDK4 further amplified the production of TNF-α, IL-1, and IL-6 following the LPS challenge, with IL-6 demonstrating the most significant increase, exceeding a 3-fold increase compared with WT males following the LPS challenge. Conversely, PDK4 knockout attenuated LPS-induced cytokine production.

In WT females, the LPS challenge significantly increased the production of all the aforementioned proinflammatory cytokines, except for IL-6. Notably, overexpression of PDK4 in females increased LPS-induced IL-6 production by over 6-fold compared with WT females. Conversely, PDK4 knockout attenuated the LPS-induced increases in cytokines. These findings suggest that PDK4 upregulation drives the production of proinflammatory cytokines, particularly IL-6, which has been identified in clinical studies and animal models as a marker associated with poorer outcomes and increased mortality in sepsis (59–61).

To further evaluate how PDK4 impacts cardiac inflammation, we also measured myeloperoxidase (MPO) activity (Figure 6F). MPO is an enzyme released by neutrophils to produce hypochlorous acid, which is a potent antimicrobial agent involved in killing bacteria and other pathogens (62, 63). In addition to its important role in the immune system’s antimicrobial defense, MPO can also cause tissue damage and is often used as a marker of inflammation (63). In both WT males and females, the LPS challenge increased MPO activity in the heart, with males showing more than 50% higher levels compared with females. Overexpression of PDK4 exacerbated this LPS-induced increase in MPO activity, while PDK4 knockout mitigated this response in both sexes. In addition, statistical analysis detected significant interactions between sex and PDK4 genotype under both sham (*P* = 0.0378) and LPS challenge (*P* = 0.0123) conditions, indicating that the effect of PDK4 on MPO levels differs between male and female hearts. Taken together, these results suggest that upregulation of PDK4 signaling contributes to cardiac inflammation, potentially through pathways of IL-6 and neutrophil infiltration.

### DCA) provides sex-specific cardiac protection during endotoxemia.

**Figure 7:**
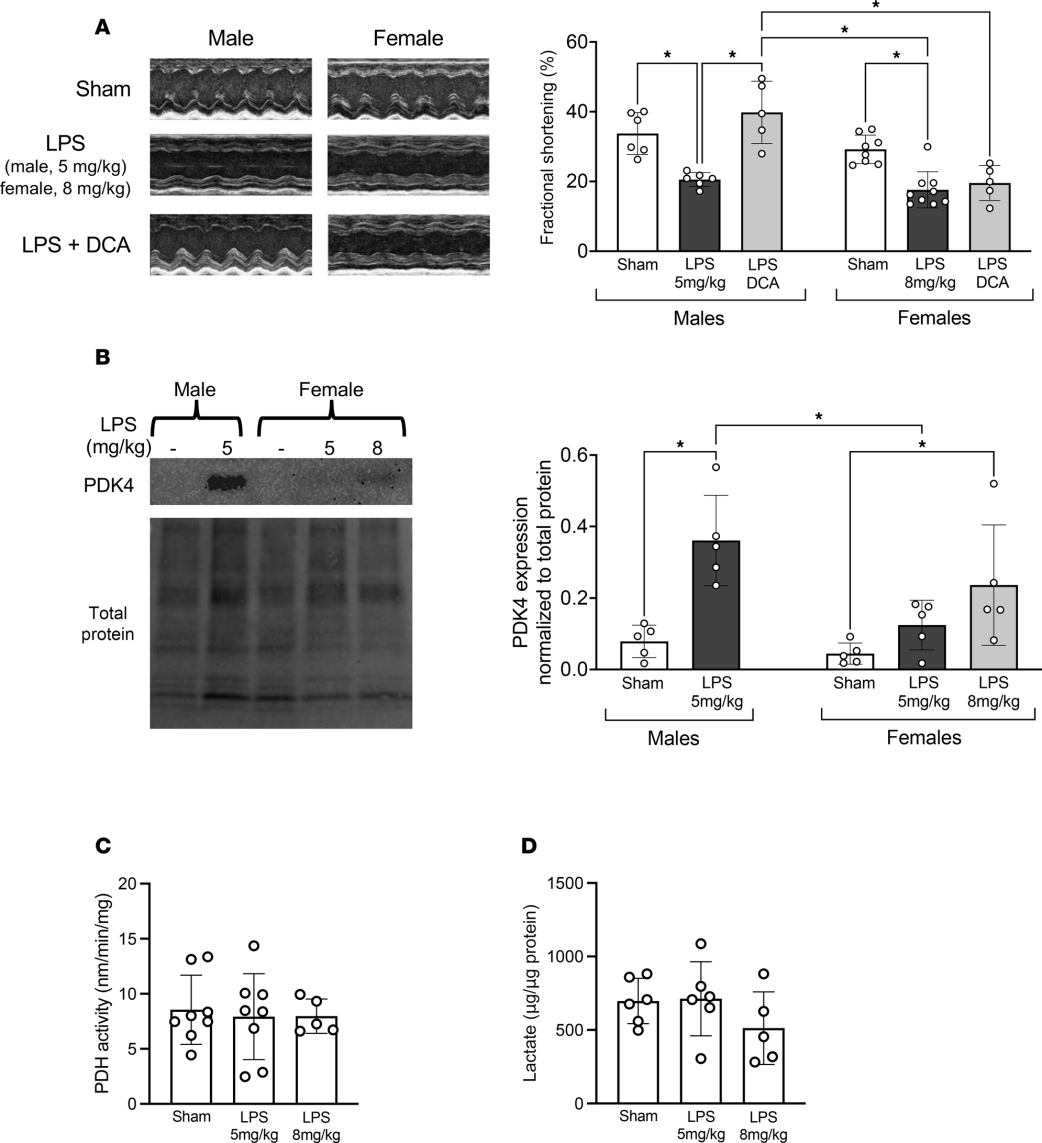
Effects of DCA on cardiac performance in endotoxemia. WT mice were given LPS challenge in doses as indicated. DCA, 25 mg/kg, was given one hour post-LPS challenge. Experiments were conducted 18 hours post-LPS challenge. (**A**) Cardiac function was assessed by echocardiography. Values of fractional shortening were compared (*n* = 5–9). (**B**) PDK4 levels in cardiac mitochondria were examined by western blot. Signals were quantified by densitometry and results were normalized to total protein (*n* = 4-5). (**C**) PDH activity and (**D**) lactate levels were measured in heart tissue lysates (*n* = 5–8 and *n* = 5–6, respectively). All data are presented as mean ± SEM and were analyzed by two-way ANOVA (*P < 0.05).

Dichloroacetate DCA is a widely used pan-PDK inhibitor that blocks PDK-mediated suppression of PDH activity (64). To explore the therapeutic potential of DCA, we tested its effects in the endotoxemia model. The dose of DCA, 25 mg/kg, was chosen based on published studies (24, 65, 66). As shown in Figure 7A, DCA treatment one hour after the LPS challenge significantly improved cardiac performance in male mice, consistent with the reported cardioprotective effects of DCA in CLP sepsis (24) and myocardial infarction (66).

In contrast with males, the effects of DCA in female mice were significantly different. Since a 5 mg/kg dose of LPS induced cardiac dysfunction in male but not female mice (Figure 1), we gradually increased the LPS dose in females to evaluate their cardiac response. We found that 8 mg/kg LPS induced cardiac dysfunction in females comparable to that observed in males at 5 mg/kg LPS. However, DCA treatment failed to ameliorate cardiac dysfunction in LPS-challenged females (Figure 7A). Statistical analysis revealed a highly significant interaction between sex and treatment (sham, LPS, or LPS plus DCA) (*P* = 0.0009), indicating that the treatment effect on cardiac performance is sex specific. Although the higher LPS dose increased PDK4 levels in cardiac mitochondrial fractions from female mice, the upregulation remained lower than in LPS-treated males (Figure 7B and Supplemental Figure 6). Interestingly, despite this stronger LPS challenge, PDH activity and lactate levels in female cardiac tissue remained largely unaffected (Figure 7, C and D, respectively). Taken together, these findings suggest that DCA-mediated protection against endotoxemia-induced cardiac dysfunction is sex specific, and they further imply that, in females, cardiac dysfunction may involve mechanisms beyond impaired glucose metabolism regulated by PDK4–PDH signaling axis.

## Discussion

Sepsis often initiates acute, overwhelming inflammation, leading to life-threatening multi-organ dysfunction, with cardiomyopathy as a significant component (1, 2, 5). Currently, sepsis treatment typically involves antibiotics and fluid resuscitation efforts, administered without consideration for potential sex-specific differences. However, emerging evidence highlights distinct physiological and immune responses between males and females during sepsis (4, 6, 7). Consequently, there is an increasing need for sex-specific approaches in sepsis management to optimize therapeutic strategies and improve patient outcomes. With a growing emphasis on precision medicine, it is imperative to develop therapeutic approaches with personalized treatment to optimize outcomes at the bedside.

Here, we used a mouse model of endotoxemia and found that acute inflammation significantly increased PDK4 expression in male compared with female hearts, with PDK4 expression being associated with sex-specific cardiac performance (Figure 1). Notably, a previously published omics study in cardiac tissue from patients with left ventricular hypertrophy similarly reported significantly higher PDK4 expression in men than in women (67), highlighting the clinical relevance for sex-biased PDK4 regulation. To investigate the mechanisms by which PDK4 regulates cardiac function, we utilized mouse models with cardiac tissue–specific PDK4 overexpression and knockout, representing gain-of-function and loss-of-function approaches, respectively. We demonstrated that PDK4 exerts sex-specific effects on myocardial metabolism (Figure 2), disrupts mitochondrial structure and dynamics (Figure 3), increases oxidative stress (Figure 4), impairs cardiac mitophagy (Figure 5), and promotes cardiac inflammation (Figure 6). Furthermore, we assessed the potential therapeutic benefit of DCA, a pan-PDK inhibitor, in improving cardiac function during endotoxemia (Figure 7). The observed sex-specific response to DCA further supports the role of PDK4 in mediating sex-specific cardiac responses during acute inflammation.

Previous studies have primarily attributed sex-specific differences to the influence of sex hormones (8, 9), with estradiol demonstrating cardioprotective effects in models of endotoxemia (68) and CLP sepsis (69). However, growing evidence suggests that sex differences are also influenced by X and Y chromosome–associated effects. A recent review highlighted sex chromosome mechanisms in cardiac development and diseases (70), emphasizing the concept that both chromosomal and hormonal factors interact to regulate cardiac physiology. In this study, our findings suggest that cardiac metabolic responses mediated through PDK4 differ between males and females under both basal conditions and during acute inflammation. Analysis of PDK4 expression in the heart tissue of FCG mice revealed male predominance (Figure 1D), suggesting contributions from sex chromosomes and long-term hormonal exposure. One limitation of this experiment is the age difference between the 17-month-old FCG mice used for PDK4 mRNA detection and the 10–12 week-old WT mice used in other experimental settings. Nonetheless, the use of these FCG mice offered valuable insight into sex chromosomes and the long-term organizational hormonal effects in a physiologically relevant context. Collectively, our data underscore the complexity of sex-specific PDK4 gene regulation and point out the need for further mechanistic studies addressing sex- and age-related differences; such studies are currently underway in our laboratory.

Cardiac metabolism plays a critical role in maintaining heart function, particularly during stress and inflammatory conditions (71–73). In this study, we observed distinct baseline metabolic profiles between male and female hearts, with males exhibiting greater glucose utilization and females exhibiting higher fatty acid utilization (Figure 2). These findings align with similar observations reported in the literature, where the underlying mechanisms may involve upregulated expression of genes in FAO pathways or enhanced fatty acid uptake in female cardiomyocytes (74). Together, these results suggest that cardiac metabolism is more complex than the traditional notion of the heart’s 60% fat and 40% glucose utilization (75). In WT male hearts, the LPS challenge reduced PDH activity and increased lactate production, consistent with the observed upregulation of PDK4 induced by LPS. Additionally, the LPS challenge decreased FAO, consistent with previous studies showing that LPS impairs fatty acid metabolism by disrupting mitochondrial function in mice (39–41). Compared with sham-treated WT males, sham-treated PDK4-Tg males showed significantly decreased PDH activity, increased lactate levels, and increased FAO, suggesting that, under physiological conditions, suppression of glucose metabolism by PDK4 upregulation leads to a metabolic shift toward fatty acid metabolism and cytosolic glycolysis. However, in PDK4-Tg male mice, the LPS challenge resulted in decreased FAO, consistent with a recent report showing that PDK4-Tg cardiomyocytes fail to compensate for deficits in glucose utilization by upregulating FAO under the stress condition of pressure overload (76). This phenomenon may result from the potential disruption of the tricarboxylic acid (TCA) cycle caused by PDK4 overexpression, which increases mitochondrial susceptibility to ROS-induced damage under stress, ultimately impairing mitochondrial function (77–80). Knockout of PDK4 in male mice significantly restored PDH activity, decreased lactate levels, and reduced FAO in response to the LPS challenge, presenting a strong resistance to the LPS challenge.

Interestingly, in the WT females, the same LPS challenge did not significantly alter PDH activity, lactate levels, or FAO, suggesting a greater capacity to maintain cardiac metabolic balance in response to inflammatory stress (Figure 2). Moreover, neither PDK4 overexpression nor knockout significantly affected PDH activity, lactate production, or FAO in sham-treated female hearts, indicating that cardiac metabolic homeostasis in females was less dependent on PDK4 signaling under normal physiological conditions. When PDK4 was transgenically overexpressed in female hearts, LPS triggered cardiac dysfunction and a significant reduction in FAO. However, lactate levels remained unaffected, suggesting the absence of a metabolic shift toward cytosolic glycolysis under this condition. This phenomenon was further supported by data showing that LPS challenge in females at a higher dose caused cardiac dysfunction and increased PDK4 expression but did not alter PDH activity or lactate levels in the cardiac tissue (Figure 7). This sex-based difference in glycolytic response may reflect variations in glucose uptake and/or utilization by cardiomyocytes, an area that warrants further investigation. Nevertheless, these findings suggest that, in males, PDK4 upregulation during acute inflammation disrupts mitochondrial energy production from both glucose and fatty acids, thereby impairing cardiac function. Supporting this, published studies have shown that activating PDH and enhancing glucose metabolism improve cardiac health and cardiac performance under stress conditions (81–83). Inhibiting PDK via DCA improves survival during endotoxemia (84) and CLP-induced sepsis (65); however, these effects in sepsis were demonstrated only using male mice. In this study, we similarly observed that DCA improved cardiac performance in male mice during endotoxemia, but not in females (Figure 7). A limitation of this experiment is that DCA is not specific to PDK4; it is a pan-PDK inhibitor (64). Additionally, long-term use of DCA raises concerns due to its potential neurotoxicity (85). While PDK4 represents a promising therapeutic target, the development of small-molecule inhibitors with greater isoform specificity and reduced toxicity is critically needed. Although PDK4-specific inhibitors are not yet available, several promising candidates are currently under development (86, 87). Nonetheless, our findings suggest that targeting PDK4 may become a promising therapeutic strategy for mitigating SIC in males, but its effectiveness may be limited in females.

Additionally, previous investigations have demonstrated that lactate has multifaceted pathological roles during sepsis. Elevated lactate levels increase vascular permeability by enhancing VE-cadherin endocytosis in endothelial cells, contributing to worsened organ dysfunction in CLP sepsis (88). In the same context, lactate also affects immune cell function by promoting the lactylation and acetylation of HMGB1 in macrophages, resulting in accelerated HMGB1 release into the extracellular environment (89). Our data show that inflammation-induced PDK4 signaling in cardiomyocytes promotes lactate production in the myocardium of males but not females. Future investigations into these lactate-mediated pathways, particularly the paracrine interactions between cardiomyocytes and other myocardial cell types, along with a focus on sex-based differences, hold significant promise for advancing our understanding of the sex-specific mechanisms underlying SIC.

In line with previously published studies (39–41), we observed that reduced FAO capacity is associated with poor cardiac performance following LPS challenge, with PDK4 exacerbating this adverse effect and promoting cardiac dysfunction in females (Figure 1C and Figure 2D). Since both male WT and PDK4-Tg mice exhibited a similar reduction in cardiac contractility in response to LPS, overexpression of PDK4 does not appear to further exacerbate endotoxemia-induced cardiac dysfunction in males. It is possible that LPS-induced upregulation of PDK4 in WT hearts already drives cardiac dysfunction to a threshold beyond which additional impairment does not occur. Consistently, a similar threshold in cardiac metabolic homeostasis was observed in PDH inhibition, lactate accumulation, and FAO impairment (Figure 2, B–D). Under baseline conditions, PDK4 overexpression in male hearts reduced PDH activity, increased lactate production, and enhanced FAO, indicating a PDK4-driven metabolic shift toward greater reliance on fatty acid utilization for energy production. This altered metabolic state appeared sufficient to maintain normal cardiac function in the absence of stress. However, upon LPS challenge, FAO was disrupted while energy demand increased due to acute inflammation, resulting in an energy deficit that precipitated cardiac dysfunction. The FAO deficiency in response to LPS may be a direct result of inflammation-induced functional deficiency in mitochondria. Additionally, PDK4 may indirectly interfere with FAO via suppression of PPARα, a key regulator of genes involved in fatty acid metabolism (90). Prior studies have shown that PPARα signaling is downregulated in the myocardium during endotoxemia (91) and CLP sepsis (92). Furthermore, a sex-based difference in PPARα expression and FAO has been reported in a mouse model of pressure overload, where genetically enhancing FAO improved myocardial energetics in females but not in males (74). A recent study identified a subset of lipid-associated macrophages that suppress FAO and increase susceptibility to sepsis (93). These findings raise the possibility that metabolic alterations in cardiomyocytes induced by PDK4 may influence the surrounding microenvironment and impact immune cell subsets, such as macrophages, within the myocardium. Investigating these mechanisms and their potential sex-specific differences represents an exciting direction for future research.

In this study, we observed and analyzed lipid droplets in the myocardium (Figure 2, E–G). Lipid droplets are dynamic organelles that store neutral lipids such as triglycerides and cholesterol esters (47). Functionally, they act as protective buffers by sequestering excess lipids and thereby preventing lipotoxicity (49). The LPS challenge increased both the number and size of lipid droplets in male and female hearts, suggesting an adaptive survival response to inflammatory stress. When PDK4 is upregulated or overexpressed, LPS reduces FAO activity, leading to a potential mismatch between fatty acid supply, utilization, and storage. It is anticipated that this imbalance could lead to an unwanted accumulation of free fatty acids, contributing to cardiac dysfunction. In contrast, myocardial ablation of PDK4 enhanced glucose metabolism and promoted the storage of fatty acids in lipid droplets, thereby providing cardiac protection. Moreover, recent studies suggest that PDK4 may regulate gene networks involved in lipid uptake, as well as lipid droplet biogenesis and turnover (94), although the direct molecular mechanisms remain to be fully elucidated. Future investigations into these regulatory pathways in the heart hold great potential for uncovering new mechanisms and identifying new therapeutic targets for SIC.

Our findings reveal that PDK4 disrupts cardiac mitochondrial structure and function during acute inflammation in a sex-specific manner, as summarized in Figures 3 and 4. Changes in cardiac metabolism are closely linked to alterations in mitochondrial structure and function (95, 96). Consistent with our previous studies (25, 28, 32, 48), the LPS challenge in WT males disrupted mitochondrial cristae structure, showing sepsis-induced mitochondrial damage in the heart. This structural disruption was accompanied by decreased mitochondrial health and increased ROS production. In contrast, WT female hearts maintained their mitochondrial cristae structure after the LPS challenge, preserved mitochondrial health, and showed no increase in ROS production, indicating greater resistance to mitochondrial damage during endotoxemia. PDK4 overexpression in both males and females resulted in disrupted mitochondrial cristae structure, reduced mitochondrial health, and elevated ROS production. Conversely, PDK4 knockout protected cardiac mitochondria and reduced oxidative stress in both sexes during endotoxemia. In males, mitochondrial dysfunction may stem from PDK4’s effects on metabolism. In females, neither PDK4 overexpression nor knockout significantly impacted glucose metabolism. However, overexpression of PDK4, combined with LPS treatment, decreased fatty acid metabolism. These findings suggest that PDK4 may have additional roles beyond its established role in regulating glucose metabolism. Interestingly, PDK4 overexpression in both sexes decreased mitochondrial size and increased mitochondrial number, whereas PDK4 knockout increased mitochondrial size and reduced mitochondrial number. These observations suggest that PDK4 upregulation promotes mitochondrial fragmentation and may also influence mitochondrial biogenesis. This conclusion is consistent with published findings that PDK4 promotes mitochondrial fragmentation, a noncanonical function beyond its role in PDH inhibition (51, 97). Excessive mitochondrial fragmentation has been linked to cardiomyopathy under various conditions, including SIC (98). In our preliminary bulk RNA-seq analysis, we did not detect a clear association between PDK4 expression and that of key mitochondrial dynamics regulators, such as fission-related proteins (DRP1 and FIS1) and fusion-related proteins (MFN1/2 and OPA1). However, it remains to be determined whether PDK4 may influence these factors through posttranslational modifications, such as phosphorylation, or through protein-protein interactions. Despite this, our current findings suggest that PDK4-mediated mitochondrial fragmentation via fission is a critical contributor to cardiac dysfunction in SIC, particularly in female hearts. Future studies will focus on elucidating the precise role of PDK4 in regulating mitochondrial network dynamics, including fission-fusion balance and mitochondrial biogenesis, during SIC.

Building on the concept that PDK4 has functions beyond its role in inhibiting PDH, we observed that PDK4 disrupts cardiac mitophagy during acute inflammation, consistent with recent findings that PDK4 upregulation interferes with the autophagy process (54, 55, 97). In our study, we investigated the cardiac mitophagy response by assessing autophagic markers associated with mitochondria and performing TEM imaging (Figure 5). Our findings revealed that WT male and female mice exhibited increased mitophagy activity in the heart following the LPS challenge. However, PDK4 overexpression significantly suppressed this mitophagy response in both sexes. Although no significant difference in LPS-induced mitophagy was observed between WT males and females, we anticipate that male hearts require a more robust mitophagy response to clear dysfunctional mitochondria due to higher levels of mitochondrial damage during inflammatory challenges. In this context, the strong upregulation of PDK4 induced by inflammation in male hearts may suppress mitophagy, resulting in the accumulation of damaged mitochondria in the myocardium. A potential mechanism for PDK4-mediated inhibition of mitophagy may involve disruption of mitochondria-associated membranes (MAMs), which leads to impaired lysosomal degradation (54). We have previously reported that LPS impairs cardiac MAMs in male mice (33). Future research is needed to elucidate PDK4’s role in regulating MAM integrity, its connections to autophagy and mitophagy, and the likelihood of sex-based differences in these processes.

Lastly, we investigated how PDK4 influences cardiac inflammation (Figure 6), given the well-established link between cardiomyocyte function, immune cell recruitment, and inflammatory responses (99, 100). The LPS challenge significantly increased TNF-α, IL-1, IL-17, and IFN-γ in both male and female mice. However, IL-6 exhibited significant sex-specific differences, with significantly higher levels observed in males compared with females following the LPS challenge. Consistent with this observation, MPO activity was also significantly higher in WT males than in females. PDK4 overexpression further amplified the production of all studied cytokines in males, with IL-6 exhibiting the largest increase, and elevated MPO activity. In females, PDK4 overexpression primarily increased IL-6 expression and significantly elevated MPO levels during endotoxemia compared with WT females. It is noteworthy that IL-6 has been shown to promote neutrophil activation and MPO release, while MPO, in turn, enhances IL-6 expression through a positive feedback loop (101–103). This interaction underscores their combined role in acute inflammation and suggests that PDK4 may contribute to this feedback mechanism, exacerbating inflammatory responses.

In summary, our findings in this report reveal a previously unrecognized role of myocardial PDK4 in mediating sex-based differences in cardiac outcomes during acute inflammatory challenges such as sepsis. Under normal conditions, PDK4 expression is low in both sexes. However, during sepsis, PDK4 is significantly upregulated in males, disrupting glucose and fatty acid metabolism, increasing ROS production, impairing mitochondrial structure and function, inhibiting mitophagy, and exacerbating cardiac inflammation, ultimately leading to heart failure. In contrast, female hearts exhibit a modest increase in PDK4 expression, enabling them to mitigate these detrimental effects and maintain functional cardiac performance. Additionally, our data also support a new role for PDK4 in regulating mitochondrial dynamics beyond its established metabolic functions. These findings highlight the complexity of cardiac metabolic regulation, underscore sex-specific mechanisms underlying SIC, and emphasize the importance of considering sex as a biological variable in the development of personalized treatments for sepsis.

## Methods

### Sex as a biological variable.

Both male and female mice were used and subject to separate statistical analyses in the experiments.

Other experimental procedures are described in the Supplemental Materials and Methods.

### Experimental animals.

WT C57BL/6J mice were obtained from Jackson Laboratory and housed at the in-campus mouse breeding core facility at Loyola University Chicago (LUC). Animals were conditioned in house for 5–6 days after arrival with commercial diet and tap water available ad libitum. The genetic mouse strain carrying cardiomyocyte-specific overexpression of PDK4 was created by engineering PDK4 gene expression under the control of cardiac-specific α-myosin heavy chain (αMHC) promoter (PDK4*-*Tg) (34). The strain with cardiomyocyte-specific inducible PDK4 knockout (PDK4-KO) was generated by crossing PDK4^fl/fl^ (Pdk4^tm1a(KOMP)Wtsi^) mice with αMHC-MerCreMer mice, which possess a tamoxifen-inducible Cre recombinase under the control of the cardiac-specific αMHC promoter (35). There were no detectable differences between the WT and PDK4^fl/fl^ mice in the physiological features and phenotypes examined in the studies. Thus, comparisons were restricted to those between the WT, PDK4-Tg, and PDK4-KO mice. The Four Core Genotypes (FCG) mice exhibited four genotypes: XXF (genetic females with female gonads), XYF (genetic males with female gonads), XXM (genetic females with male gonads), and XYM (genetic males with male gonads) (36).

### Mouse model of endotoxemia.

Endotoxemia was induced in mice aged 8–12 weeks using lipopolysaccharide (LPS) (Millipore-Sigma; L3012) (28, 31, 32). LPS was administered intraperitoneally based on the individual body weight to ensure precise dosing. Sham groups received sterile, endotoxin-free PBS. The pan-PDK inhibitor dichloroacetate (DCA) (Millipore-Sigma; 347795) was administered intraperitoneally at 25 mg/kg, one hour after the LPS challenge in related experiments.

### Statistics.

All data are presented as mean ± SEM. Comparisons between 2 groups were analyzed using a 2-tailed unpaired Student’s *t* test. For comparisons among multiple groups, 2-way or 3-way ANOVA was used as appropriate, including evaluation of interactions among variables. A *P*-value ≤ 0.05 was considered statistically significant.

### Study approval.

All animal procedures were approved by the Institutional Animal Care and Use Committee (IACUC) at Loyola University Chicago and were conducted following institutional guidelines and the National Research Council’s “Guide for the Care and Use of Laboratory Animals”.

### Data availability.

Data in this study are available within the article and its supplemental files. Additional results generated and analyzed during the study are available from the corresponding author upon reasonable request. Values for all data points in graphs are reported in the Supporting Data Values file.

## Author contributions

QSZ, JQY, and AN conceived of the project. JQY, AN, MK, QC, DJR, JEL, AA, LYZ, YS, and BR carried out the experiments. JQY, AN, LYZ, JEL, AA, SE, HLJ, XG, QD, and QSZ analyzed the data. JQY, AN, DJR, LS, SE, QD, and QSZ wrote and revised the manuscript. LS, HS, SE, and MKG acquired reagents and animal models. All authors have read and agreed to the published version of the manuscript.

## Supplementary Material

Supplemental data

Unedited blot and gel images

Supporting data values

## Version 1 07/08/2025

Electronic publication
