# Supplementary material for: Myocardial pyruvate dehydrogenase kinase 4 drives sex-specific cardiac responses to endotoxemia
Source: JCI Insight. 2025 Jul 8;10(13):e191649. doi: 10.1172/jci.insight.191649 (PMC12288905; doi:10.1172/jci.insight.191649)
Supplement: Supplemental data [file jciinsight-10-191649-s171.pdf]

Supplemental Materials and Methods

Tamoxifen administration for PDK4 knockout – According to the published protocol (1), PDK4 knockout was achieved by homologous recombination following administration of tamoxifen (20 mg/kg) via intraperitoneal injection every other day for a total of three doses over a 5-day period, beginning two weeks before experimentation. Tamoxifen (Millipore-Sigma, Burlington, MA; catalog number T5648) was dissolved in a solution of 90% sesame oil and 10% ethanol to prepare the stock solution, which was stored at –20°C. Before injection, the solution was warmed to 55°C for 30 minutes.

Echocardiography **–** Transthoracic echocardiograms were recorded in sedated mice using the Visual Sonics Vevo 3100 small animal echocardiography machine, following the established protocol (2-4). Views were captured in planes that closely resembled the parasternal short-axis view (M-mode) and the apical long-axis view (B-mode), with approximately 2 sets of data acquired from each animal, each lasting around 5 seconds. The mice were anesthetized with isoflurane (3-5% for induction and 1-1.5% for maintenance) and maintained at a temperature of 37°C throughout the procedure. Care was taken to ensure that the heart rate of the mice fell within the range of 350-500 beats per minute. Subsequently, cardiac function was computed offline utilizing the VisualSonics VevoLab cardiography software packages.

Preparation of total tissue lysates and mitochondrial fractions **–** Heart tissues were harvested, washed in PBS, snap clamped frozen, and kept at -80°C. Tissue lysates were prepared using tissue protein extraction reagent (T-PER) (Thermo Fisher Scientific, Rockford, IL; catalog

number 78510), supplemented with protease inhibitors, according to the manufacturer's protocol. In some experiments, crude mitochondrial fractions were isolated using a mitochondrial isolation kit (Thermo Fisher Scientific, Waltham, MA; catalog number 89801). To obtain a relatively pure pool of mitochondria, the crude mitochondrial fractions were homogenized with T-PER lysis buffer containing protease inhibitors by incubation on ice for 30 minutes with gentle vortexing every 10 minutes. The samples were then centrifuged at 12,000g, and the supernatants were collected. Protein concentrations in all preparations were quantified using a detergent-compatible Bradford assay kit (Thermo Fisher Scientific, Waltham, MA; catalog number 23246).

Real-time quantitative PCR (RT-qPCR) – Frozen tissue samples were preserved by immersion in a prechilled RNAlater-ICE Frozen Tissue Transition Solution (Thermo Fisher Scientific, Waltham, MA; catalog number AM7030) and then stored at -20°C overnight. Total RNA was then extracted using the TRIzol reagent-chloroform method (Thermo Fisher Scientific, Waltham, MA; catalog number 15596018) following the manufacturer's instructions. The isolated RNA samples were resuspended in RNase-free water, and their quantity and quality were assessed using a NanoDrop. RNA integrity was evaluated by denaturing agarose gel electrophoresis, in which strong signals of 28S rRNA and 18S RNA were revealed and their approximate ratio was 2:1. For quantitative PCR (qPCR), gene-specific primers were retrieved from PrimerBank (http://pga.mgh.harvard.edu/primerbank/) (5) and synthesized at the MGH DNA Core facility (https://dnacore.mgh.harvard.edu/synthesis/index.shtml). For PDK4 detection, the 5' forward primer sequence is CCGCTGTCCATGAAGCA, and the 3' reverse primer sequence is GCAGAAAAGCAAAGGACGTT. All primers underwent desalting and were subjected to quality

assessment through both UV absorbance and capillary electrophoresis. Reverse transcription was carried out using the SuperScript First-Strand Synthesis System for RT-PCR (Thermo Fisher Scientific, Waltham, MA; catalog number 11904018) by following the manufacturer's protocol. Real-time PCR experiments were then conducted on an ABI Prism SDS 7000 instrument with the following program: (1) Initial denaturation at 95°C for 10 minutes; (2) 40 cycles of denaturation at 95°C for 15 seconds, annealing at 60°C for 30 seconds, and extension at 72°C for 30 seconds; and (3) a final extension at 72°C for 10 minutes. The results were subsequently analyzed using the built-in SDS 7000 software.

Western blotting – Procedures were performed according to an established protocol (2). Briefly, prepared SDS-PAGE protein samples were loaded and run on 4-15% SDS-PAGE gels and transferred to PVDF membranes. Membranes were blocked with 5% nonfat milk-PBS at room temperature for 1 hour and subsequently probed with respective primary antibodies. The membranes were then rinsed and incubated with horseradish peroxidase-conjugated anti-rabbit IgG (Bio-Rad, Hercules, CA; catalog number 170-6515). Antibody dilutions and incubation time were determined by the vendor’s instructions. After washing off excess secondary antibody, membranes were rinsed, and bound antibodies were detected by using Pierce ECL Western Blotting Substrate (Thermo Scientific, Waltham, MA; catalog number 32106). Primary antibodies used in this study include anti-LC3A/B (Cell Signaling, Danvers, MA; catalog number 4108), anti-p62 (Cell Signaling, Danvers, MA; catalog number 5114) and anti-PDK4 (custom-generated by Biosynthesis, Inc., targeting a peptide sequence mixture of CIPSREPKNLAKE-KLA, DLVEFHEKSPEDQKALSE, and EFVDTLVKVRNRHH-NVVPT, as previously described (6)).

Measurement of pyruvate dehydrogenase (PDH) activity **–** PDH activity in heart tissue lysates were measured using a PDH assay kit (MilliporeSigma, Burlington, MA; catalog number MAK183) according to the vendor's instructions. Briefly, 50mg of heart tissue was homogenized in 250ul of PDH assay buffer. Samples were centrifuged at 10,000 x g for 10 minutes at 4°C to remove insoluble material and supernatant was collected. Samples were diluted 1:2 in PDH assay buffer and 50ul of sample and 50ul of reaction mix were loaded onto a 96 well plate and incubated at 37°C protected from light. The initial absorbance was measured at 450 nm using a plate reader and the final absorbance was measured after 15 minutes. Results were normalized by the amount of protein in tissue lysate samples, and all samples were run in triplicate.

Quantification of lactate levels **–** The levels of lactate in heart tissue lysates were measured using a lactate assay kit (MilliporeSigma, Burlington, MA; catalog number MAK064) according to the vendor's instructions. Briefly, 50mg of heart tissue was homogenized in 250ul of lactate assay buffer. Samples were centrifuged at 13,000 x g for 10 minutes to remove insoluble material and supernatant was collected. Samples were diluted 1:2 in lactate assay buffer and 50ul of sample and 50ul of reaction mix were loaded onto a 96 well plate and incubated for 15 minutes at room temperature, protected from light. The absorbance was measured at 570 nm using a plate reader. Results were normalized by the amount of protein in tissue lysate, and all samples were run in triplicate.

Quantification of malondialdehyde (MDA) levels **–** The levels of MDA in heart tissue lysates were obtained using a lipid peroxidation assay kit (Abcam, Cambridge, MA; catalog number ab118970) according to the vendor's instructions. Briefly, 10mg of heart tissue was

homogenized in 100ul of MDA lysis buffer. Samples were centrifuged at 13,000 x g for 10 minutes to remove insoluble material and supernatant was collected. Developer VII/TBA reagent was added into each sample. Samples were incubated at 95°C for 60 minutes and cooled to room temperature in an ice bath for 10 minutes. Samples were loaded onto a 96 well plate and absorbance was measured at 532 nm using a microplate reader. Results were normalized by the amount of protein in tissue lysate samples, and all samples were run in triplicate.

Measurements of cytokines by enzyme-linked immunosorbent assay (ELISA) **–** Cytokine levels in total heart lysates were measured using Bio-Plex Mouse Cytokine Panel A 6-Plex (Bio-Rad, Hercules, CA; catalog number M6000007NY) according to the vendor's instructions. Results were normalized by the amount of protein for tissue lysates, and all samples were run in triplicate.

Isolation of primary cardiomyocytes from mouse hearts **–** Adult cardiomyocytes were isolated as previously described (7). Briefly, animals were anesthetized with isoflurane. The aorta was clamped *in-situ*, after which the heart was excised and anterogradely perfused with a digestion solution containing collagenase 2 (Worthington, Lakewood, NJ; catalog number LS004176), collagenase 4 (Worthington, Lakewood, NJ; catalog number LS004188) and protease XIV (MilliporeSigma, Burlington, MA; catalog number P5147). After digestion, cardiac tissue was dissociated and filtered through a 100 µm mesh. Cardiomyocytes were left to settle at room temperature for 15 minutes and resuspended in calcium re-introduction buffers (51). After allowing the cells to pellet in the last calcium re-introduction buffer, cells were resuspended in

plating media (51), seeded onto PDL (MilliporeSigma, Burlington, MA; catalog number P6407) coated 35mm glass-bottom petri dishes and left to adhere at 37^{0}C for 20 minutes.

Fluorescence Microscopy **–** Cells were washed twice with Tyrode buffer (7). Imaging was then performed using an LSM 510 confocal microscope, which features an Axio Observer Z1 motorized inverted microscope and Zen software (Carl Zeiss Microscopy), to capture images of the cells at 10× magnification. JC-1 (Thermo Scientific, Waltham, MA; catalog number T3168) fluorescence was excited at 560 nm and at 485 nm, and images were acquired with an exposure time of 1,000 ms as previously described (51). MitoSOX (Thermo Scientific, Waltham, MA; catalog number M36008) fluorescence was excited at 510 nm, and images were acquired with an exposure time of 10,000 ms as previously described (51). Fluorescence of the cell was analyzed using ImageJ (7).

Transmission Electron microscopy (TEM) **–** TEM was performed as previously described (7). Briefly, cardiomyocytes were washed in PBS and immersed in PBS containing 2% paraformaldehyde, 2.5% glutaraldehyde, and 0.2% tannic acid for 1 hour at room temperature. Samples were then fixed, stained *en bloc* via immersion in 1% uranyl acetate dissolved in 50% ethanol, dehydrated via immersion in an ascending series of alcohols (25%, 50%, 75%, 95%, 100% alcohol), and incubated in propylene oxide. Cells were then incubated in a 1:1 ratio of propylene oxide to epoxy resin. Ultrathin sections (90 nm) were cut with an ultramicrotome (EM UC7, Leica Microsystems), mounted on formvar- and carbon-coated 200 mesh copper grids and stained with filtered 1% uranyl acetate and Reynold's lead citrate prior to imaging. Samples were imaged using a Philips CM 120 transmission electron microscope (TSS Microscopy) equipped

with a BioSprint 16-megapixel digital camera (Advanced Microscopy Techniques). Images were analyzed using ImageJ, and cristae disorganization was determined by the appearance of swollen, fragmented, or irregularly shaped structures according to our established protocol (7).

Measurement of fatty acid oxidation **–** Cardiomyocytes were resuspended in 5ml of M199 culture media (Thermo Scientific, Waltham, MA; catalog number 11043023) and allowed to settle at room temperature for 15 minutes. After 15 minutes, M199 was removed, and cardiomyocytes were resuspended in 1ml of M199. Cells were counted by a hemocytometer and then incubated with 10uM FAO blue dye in M199 and 50mM of BSA conjugated palmitate. 3000 cells/well were loaded into a black 96-well plate in triplicates and incubated at 37^{0}C protected from light. The initial absorbance was measured at 420/465 excitation and emission using a SpectraMax iD5 plate reader (Molecular Devices, San Jose, CA), reading bottom to top, and the final absorbance was measured after 20 minutes. Fatty acid oxidation was calculated as the change in fluorescence after 20 minutes.

Supplement Figures

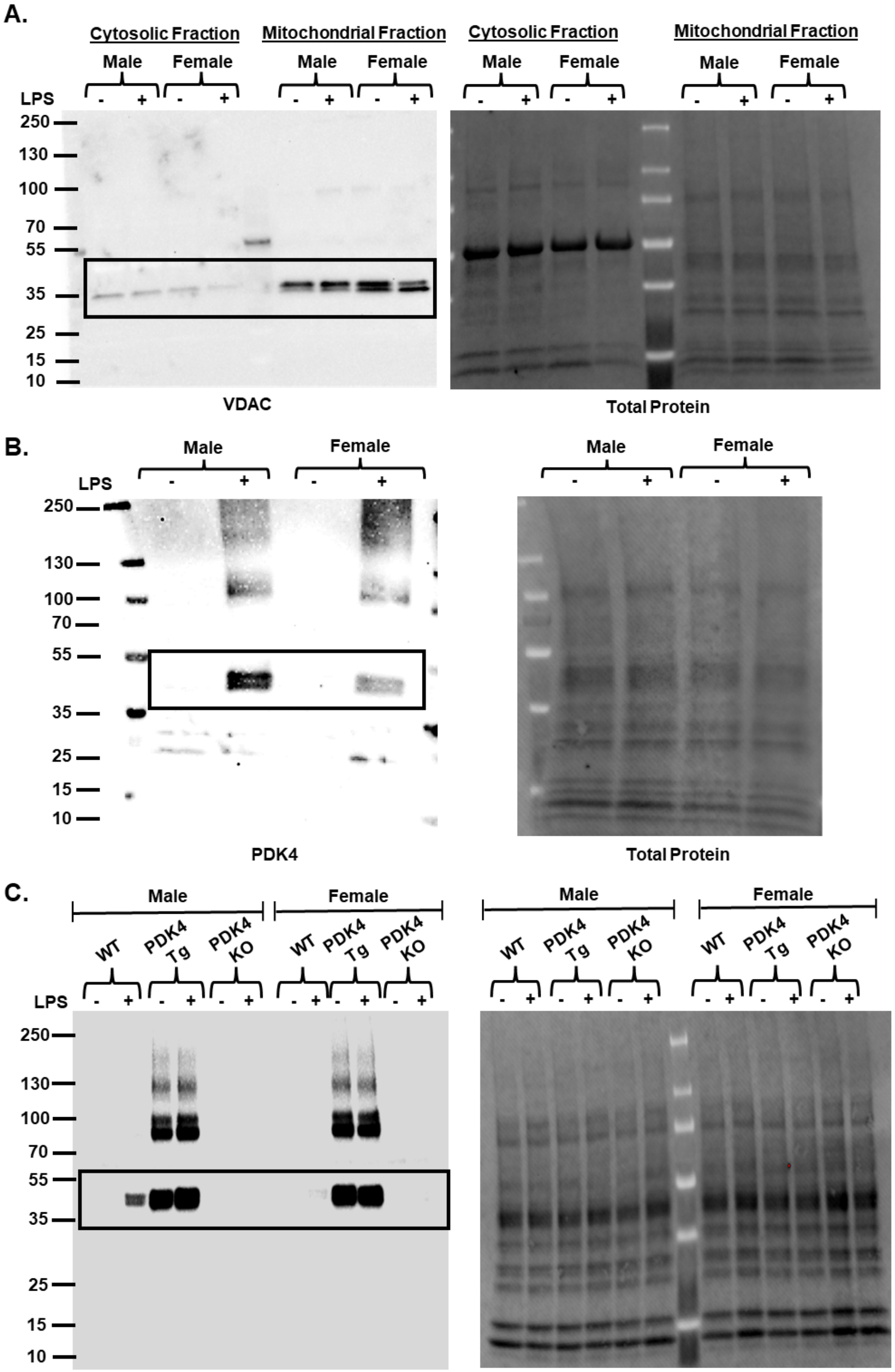

Supplemental Figure S1: Uncropped western blots for VDAC and PDK4. A) Uncropped western blot showing VDAC in cytosolic and mitochondrial fractions from the heart tissue of WT male and female mice with the LPS challenge or sham treatment. **B)** Uncropped western blot corresponding to Figure 1A. **C)** Uncropped western blot showing PDK4 in cardiac tissue lysates from WT, PDK4-Tg and PDK4-KO male and female mice with the LPS challenge or sham treatment.

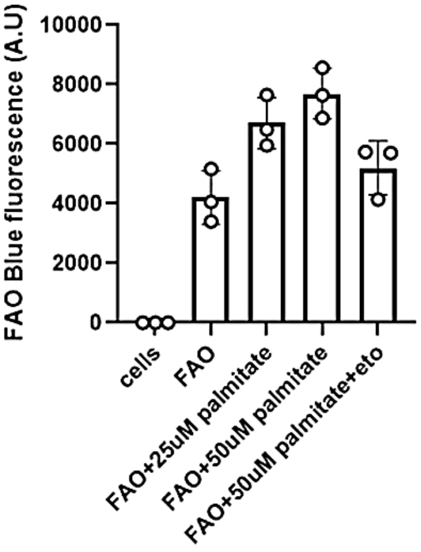

Supplemental Figure S2: Validation of quantification of FAO activity using FAO blue. FAO blue fluorescence in cardiomyocytes under different conditions: no dye negative control, FAO blue, FAO plus 25 µM palmitate, FAO plus 50 µM palmitate, and FAO plus 50 µM palmitate and 40 µM etomoxir, a FAO inhibitor (N=3 independent isolations per group).

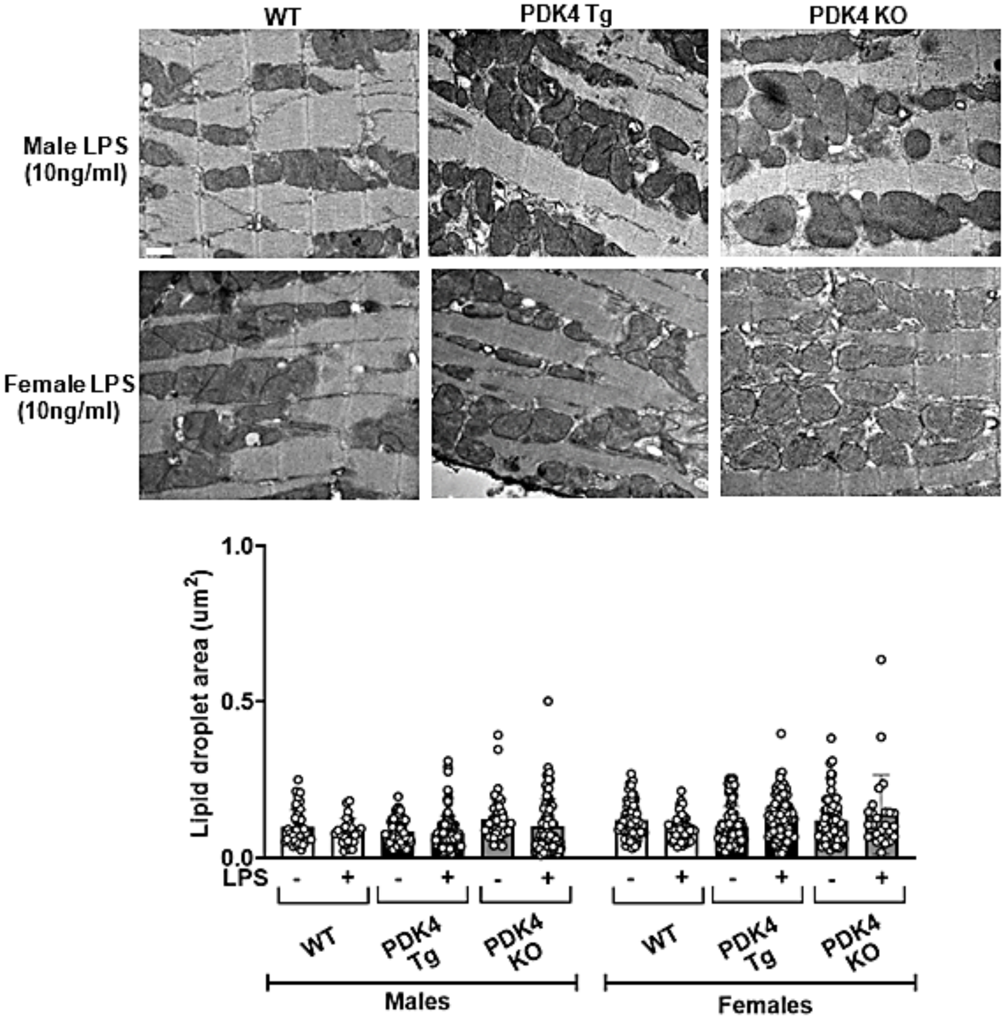

Supplemental Figure S3. *In vitro* LPS treatment does not stimulate lipid droplet formation in cardiomyocytes. Representative TEM images of cardiomyocytes following in vitro LPS treatment (10 ng/mL for 1 hour). Images are representative of 2-3 independent cardiomyocyte isolations per group. Scale bar: 1um (applies to all panels). Lipid droplet area was quantified based on TEM images using Image J software (N=26-137 lipid droplets per group).

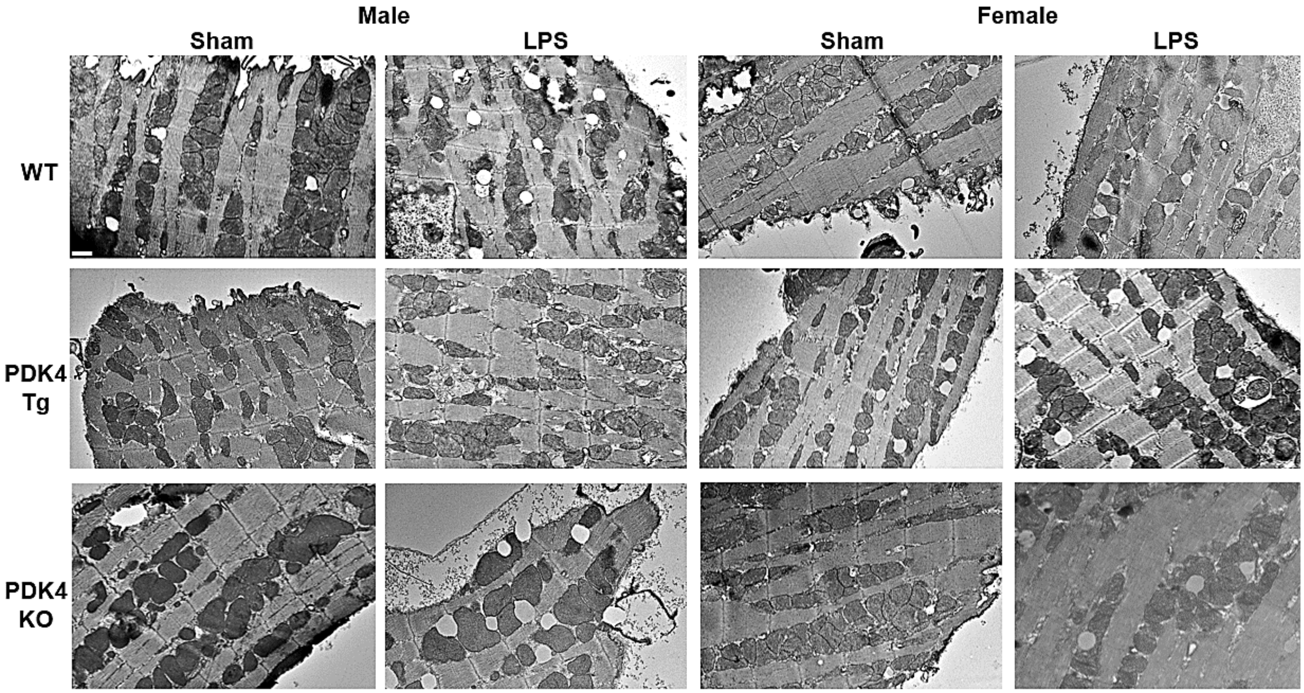

Supplemental Figure S4: Lower magnification TEM images corresponding to Figure 2E. Representative TEM images of cardiomyocytes from LPS challenged or sham-treated WT, PDK4-Tg or PDK4 KO mice. Images are representative of 3 independent cardiomyocyte isolations per group. Scale bar: 1um (applies to all panels).

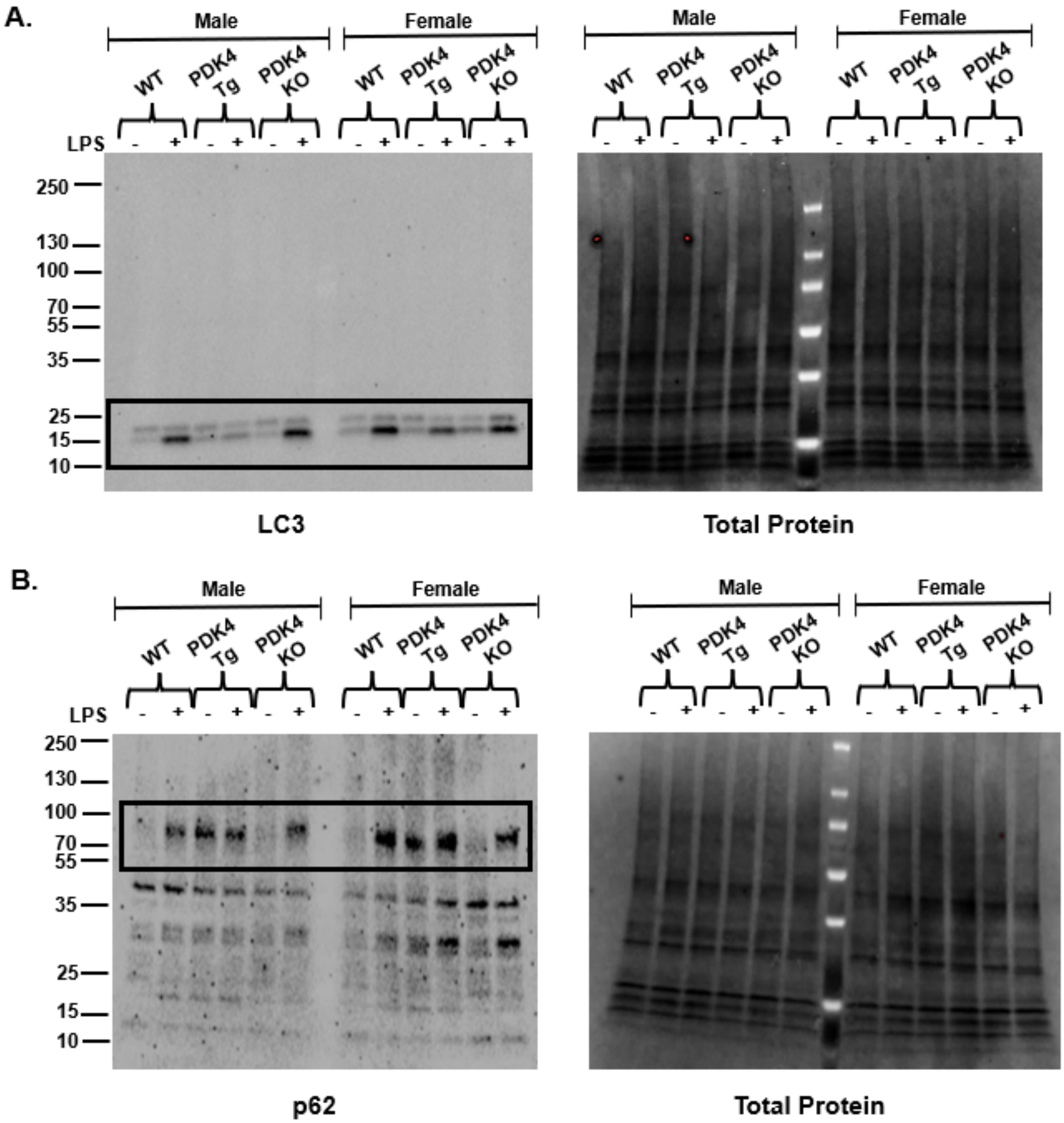

Supplemental Figure S5: Uncropped western blots for LC3 and p62. Uncropped western blot corresponding to Figures 5 A and B.

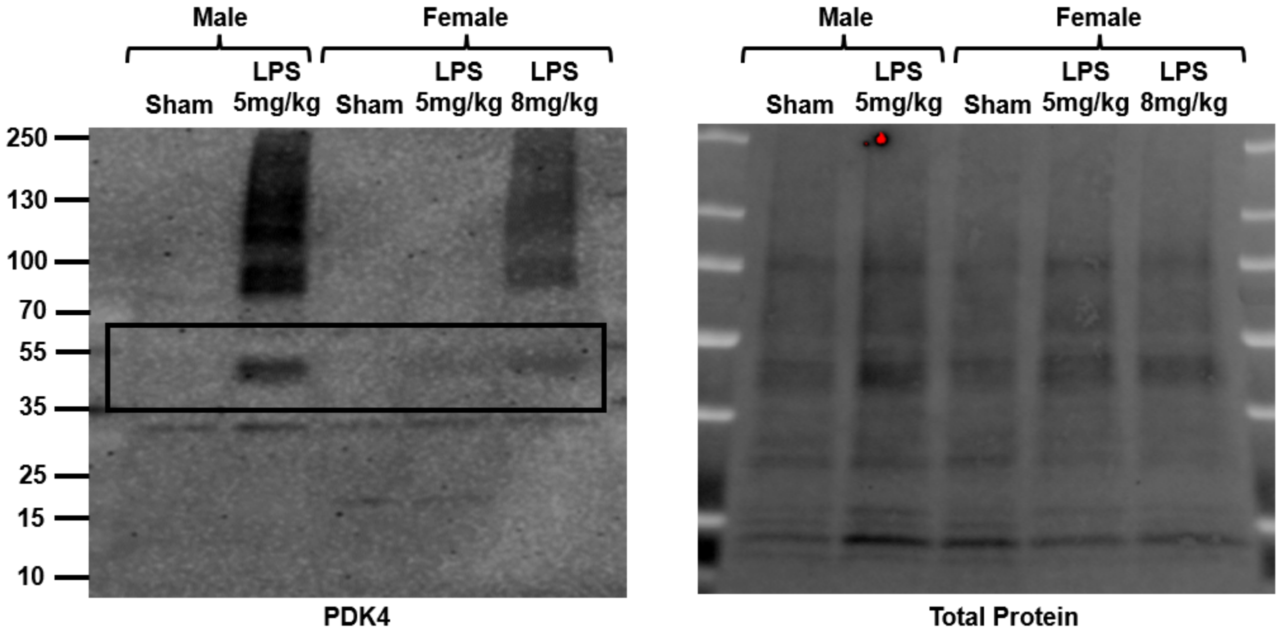

Supplemental Figure S6: Uncropped western blots for PDK4. Uncropped western blot corresponding to Figure 7B.

References

1. Cardoso AC, Lam NT, Savla JJ, Nakada Y, Pereira AHM, Elnwasany A, et al. Mitochondrial Substrate Utilization Regulates Cardiomyocyte Cell Cycle Progression. *Nat Metab.* 2020;2(2):167-78.
2. Sun Y, Yao X, Zhang QJ, Zhu M, Liu ZP, Ci B, et al. Beclin-1-Dependent Autophagy Protects the Heart During Sepsis. *Circulation.* 2018;138(20):2247-62.
3. Gao S, Ho D, Vatner DE, and Vatner SF. Echocardiography in Mice. *Curr Protoc Mouse Biol.* 2011;1:71-83.
4. Li X, Liu J, Hu H, Lu S, Lu Q, Quan N, et al. Dichloroacetate Ameliorates Cardiac Dysfunction Caused by Ischemic Insults Through AMPK Signal Pathway-Not Only Shifts Metabolism. *Toxicol Sci.* 2019;167(2):604-17.
5. Wang X, and Seed B. A PCR primer bank for quantitative gene expression analysis. *Nucleic Acids Res.* 2003;31(24):e154.
6. Crewe C, Schafer C, Lee I, Kinter M, and Szweda LI. Regulation of Pyruvate Dehydrogenase Kinase 4 in the Heart through Degradation by the Lon Protease in Response to Mitochondrial Substrate Availability. *J Biol Chem.* 2017;292(1):305-12.
7. Nikouee A, Yap JQ, Rademacher DJ, Kim M, and Zang QS. An optimized Langendorff-free method for isolation and characterization of primary adult cardiomyocytes. *BMC Cardiovasc Disord.* 2024;24(1):649.
