# Supplementary material for: Myocardial pyruvate dehydrogenase kinase 4 drives sex-specific cardiac responses to endotoxemia
Source: JCI Insight. 2025 Jul 8;10(13):e191649. doi: 10.1172/jci.insight.191649 (PMC12288905; doi:10.1172/jci.insight.191649)
Supplement: Unedited blot and gel images [file jciinsight-10-191649-s172.pdf]

Supplement Gel Images (Included in Supplemental Materials)

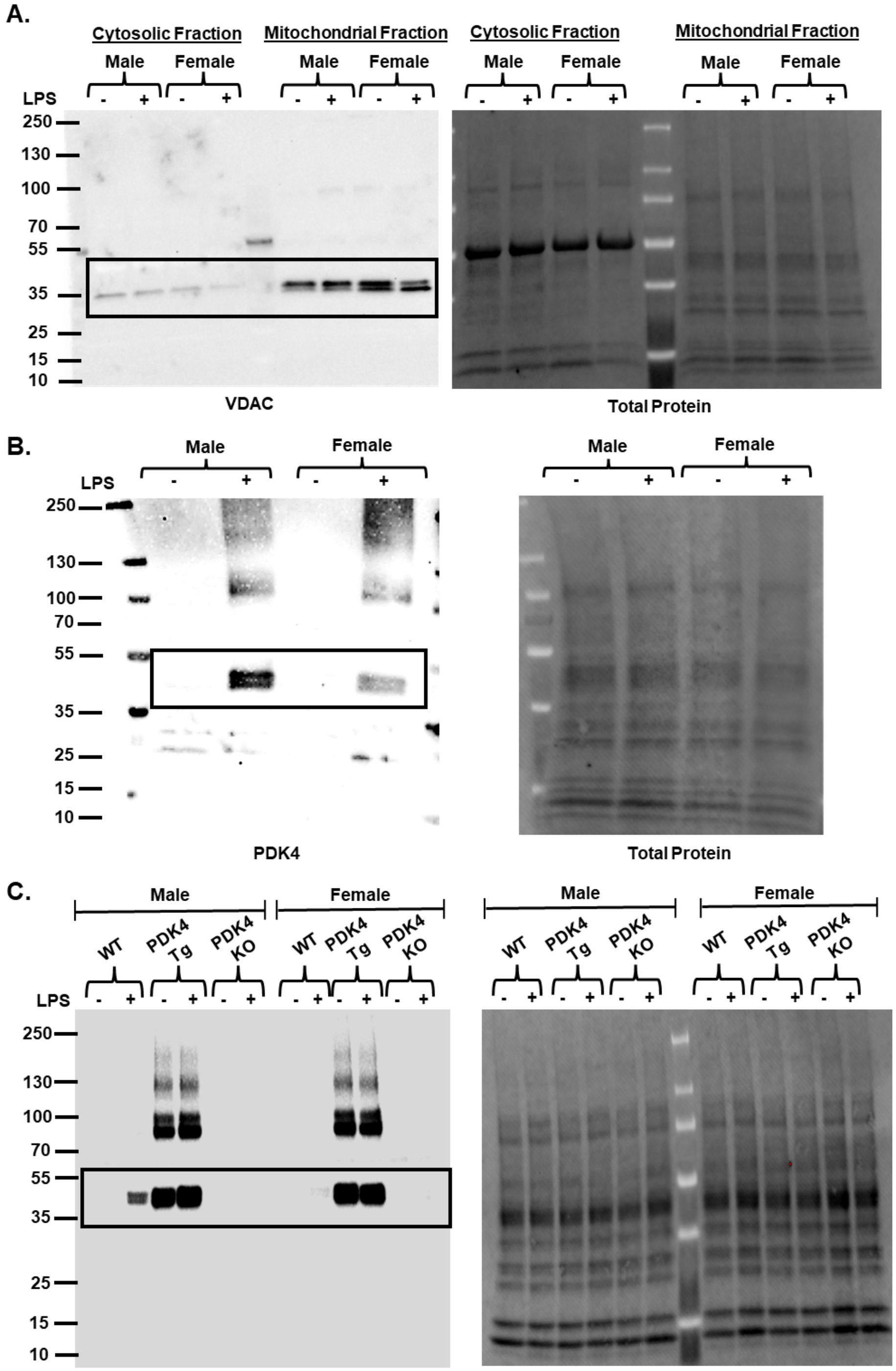

Supplemental Figure S1: Uncropped western blots for VDAC and PDK4. A) Uncropped western blot showing VDAC in cytosolic and mitochondrial fractions from the heart tissue of WT male and female mice with the LPS challenge or sham treatment. **B)** Uncropped western blot corresponding to Figure 1A. **C)** Uncropped western blot showing PDK4 in cardiac tissue lysates from WT, PDK4-Tg and PDK4-KO male and female mice with the LPS challenge or sham treatment.

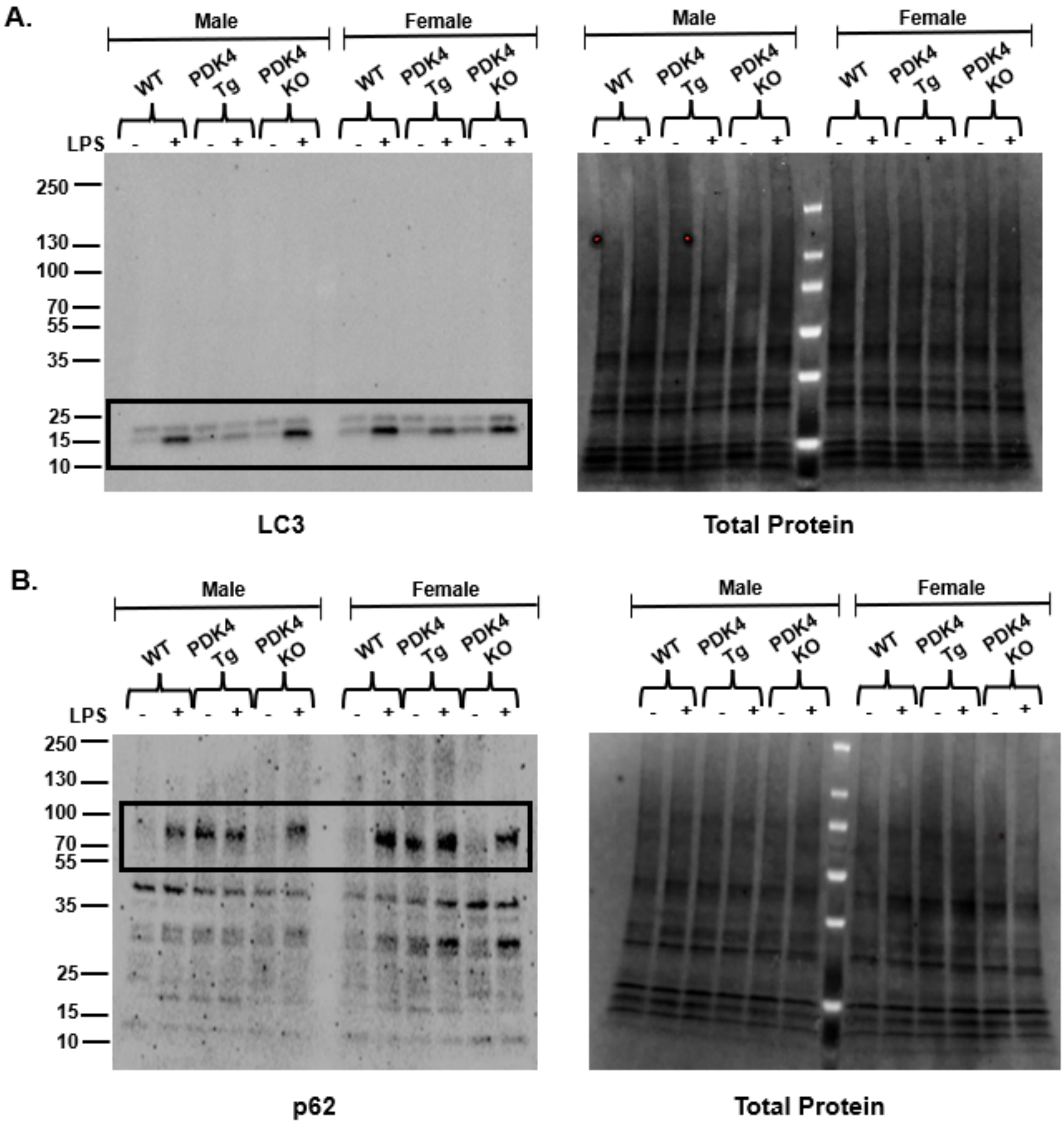

Supplemental Figure S5: Uncropped western blots for LC3 and p62. Uncropped western blot corresponding to Figures 5 A and B.

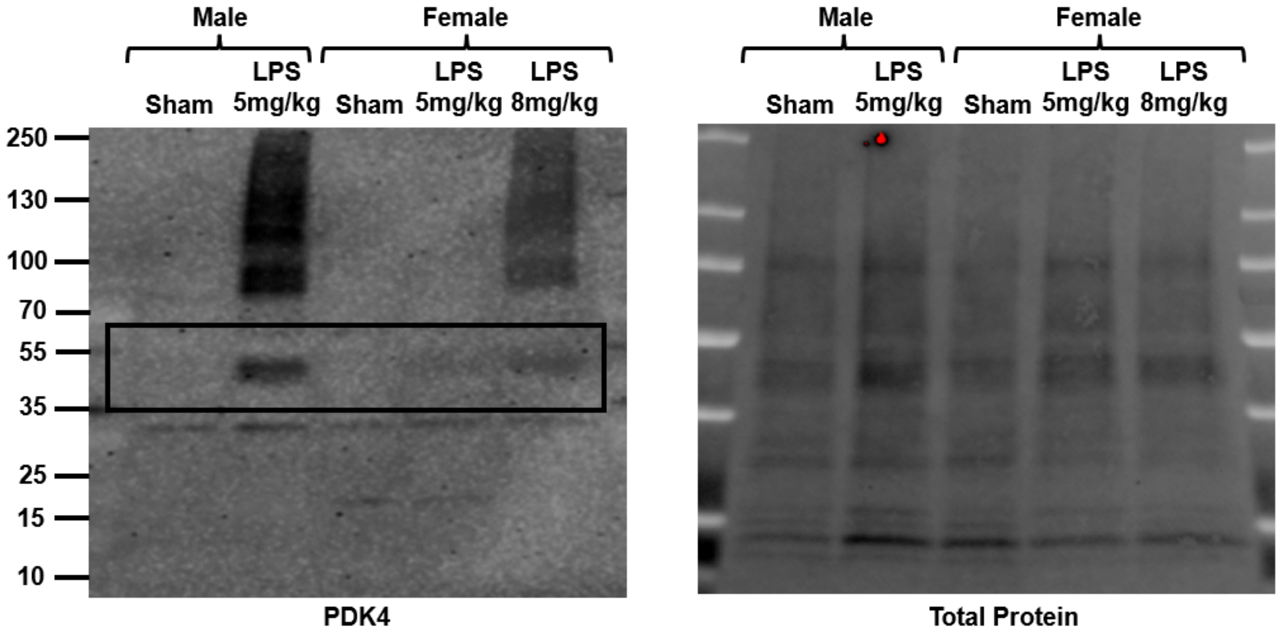

Supplemental Figure S6: Uncropped western blots for PDK4. Uncropped western blot corresponding to Figure 7B.
